# miR-378 influences muscle satellite cells and enhances adipogenic potential of fibro-adipogenic progenitors but does not affect muscle regeneration in the glycerol-induced injury model

**DOI:** 10.1038/s41598-023-40729-x

**Published:** 2023-08-18

**Authors:** Olga Mucha, Paulina Podkalicka, Monika Żukowska, Ewelina Pośpiech, Józef Dulak, Agnieszka Łoboda

**Affiliations:** 1 Department of Medical Biotechnology, Faculty of Biochemistry, Biophysics and Biotechnology, Jagiellonian University in Krakow, Gronostajowa 7, 30-387 Kraków, Poland; 2 Malopolska Centre of Biotechnology in Krakow, 30-387 Kraków, Poland

**Keywords:** Mechanisms of disease, Regenerative medicine

## Abstract

Skeletal muscle regeneration relies on the reciprocal interaction between many types of cells. Regenerative capacity may be altered in different disorders. In our study, we investigated whether the deletion of miR-378a (miR-378) affects muscle regeneration. We subjected 6-week-old wild-type (WT) and miR-378 knockout (miR-378^–/–^) animals to the glycerol-induced muscle injury and performed analyses in various time-points. In miR-378^–/–^ animals, an elevated abundance of muscle satellite cells (mSCs) on day 3 was found. Furthermore, fibro-adipogenic progenitors (FAPs) isolated from the muscle of miR-378^–/–^ mice exhibited enhanced adipogenic potential. At the same time, lack of miR-378 did not affect inflammation, fibrosis, adipose tissue deposition, centrally nucleated fiber count, muscle fiber size, FAP abundance, and muscle contractility at any time point analyzed. To conclude, our study revealed that miR-378 deletion influences the abundance of mSCs and the adipogenic potential of FAPs, but does not affect overall regeneration upon acute, glycerol-induced muscle injury.

## Introduction

Skeletal muscles are not only a well-organized structural unit of the human body, but also very precise, adaptive, and dynamic in terms of remodeling and regeneration machinery. This plasticity is required as muscles are constantly exposed to various physiological and pathological perturbations, including changes in metabolic demands, thermal stress, exercise, aging, and injury^1,2^. To preserve, remodel, and/or restore structure and functionality after injury, skeletal muscles developed a highly organized regenerative mechanism, involving a reciprocal interaction between many types of cells^3,4^. In response to injury and appropriate external and internal signals, muscle satellite cells (mSCs) are activated, proliferate, differentiate, and finally fuse, giving birth to newly formed muscle fibers. Other muscle-resident cells are responsible for the creation of a supportive niche for efficient regeneration. These cells include immune cells (such as M1 and M2 macrophages), fibroblasts, fibro-adipogenic progenitors (FAPs), and many others^5,6^. The precise regulation of cellular interactions and their time separation is required, and any dysregulation could lead to insufficient regeneration, accumulation of fibrous tissue, and weakening of the muscle.

Critical regulators of muscle regeneration are microRNAs acting on the activation, proliferation, ad differentiation of mSCs.

miR-378a (miR-378) is a small non-coding miRNA incorporated into the first intron of the peroxisome proliferator-activated receptor-gamma coactivator 1-beta gene (*PPARGC1b*) encoding PGC1β, a primary regulator of mitochondrial biogenesis, lipid, and glucose metabolism. miR-378 is highly expressed in brown adipose tissue, heart muscle, and skeletal muscles^7^. The abundance of miR-378 in skeletal muscles was also confirmed in our previous studies^8,9^. miR-378 was suggested to serve as a potential biomarker for various conditions, including Duchenne muscular dystrophy (DMD), an incurable, genetic disorder caused by the lack of dystrophin–structural protein in muscles. The level of miR-378 was upregulated in serum from patients and mouse models of DMD^9,10^. We previously demonstrated better physical performance of miR-378-deficient *mdx* mice (commonly used DMD mouse model), with significantly reduced inflammation and fibrosis (with a decrease in the number of FAPs), and a lower percentage of regenerating fibers^9^. Furthermore, our recent study revealed that loss of miR-378 results in the modulation of systemic metabolism in *mdx* mice, including improved glucose tolerance, restored liver glycogen content, and altered expression of genes related to lipogenesis and lipid storage^11^.

Taking into account the literature indications regarding the role of miR-378 in, among others, muscle biology, and our previous observations in the DMD model, the aim of the present study was to research the effect of miR-378 deletion on muscle regeneration utilizing a glycerol-induced injury model^9,11^. Intramuscular injection of glycerol is an acute muscle injury model in which degenerative changes resemble those seen in dystrophy^12,13^, but various stages can be studied separately and in a more controlled manner. We mainly focused on the FAP-related processes, including fibrosis and adipogenesis. miR-378 is well known for its role in lipid metabolism^14^, and FAPs can differentiate not only into the extracellular matrix (ECM)-producing fibroblasts, but also adipocytes^15^.

Our results demonstrated that the lack of miR-378 increased the number of mSCs, and favored the adipogenic potential of isolated FAPs shortly after the damage. However, it did not affect inflammation, fibrosis, adipose tissue deposition, centrally nucleated fiber count, muscle fiber size, FAP abundance, and, most importantly, muscle contractile properties. Hence, although miR-378 deficiency influenced the response to glycerol-induced muscle injury, it did not compromise muscle regeneration.

## Results

### Lack of miR-378 influences muscle damage markers but does not affect muscle functionality

**Figure 1:**
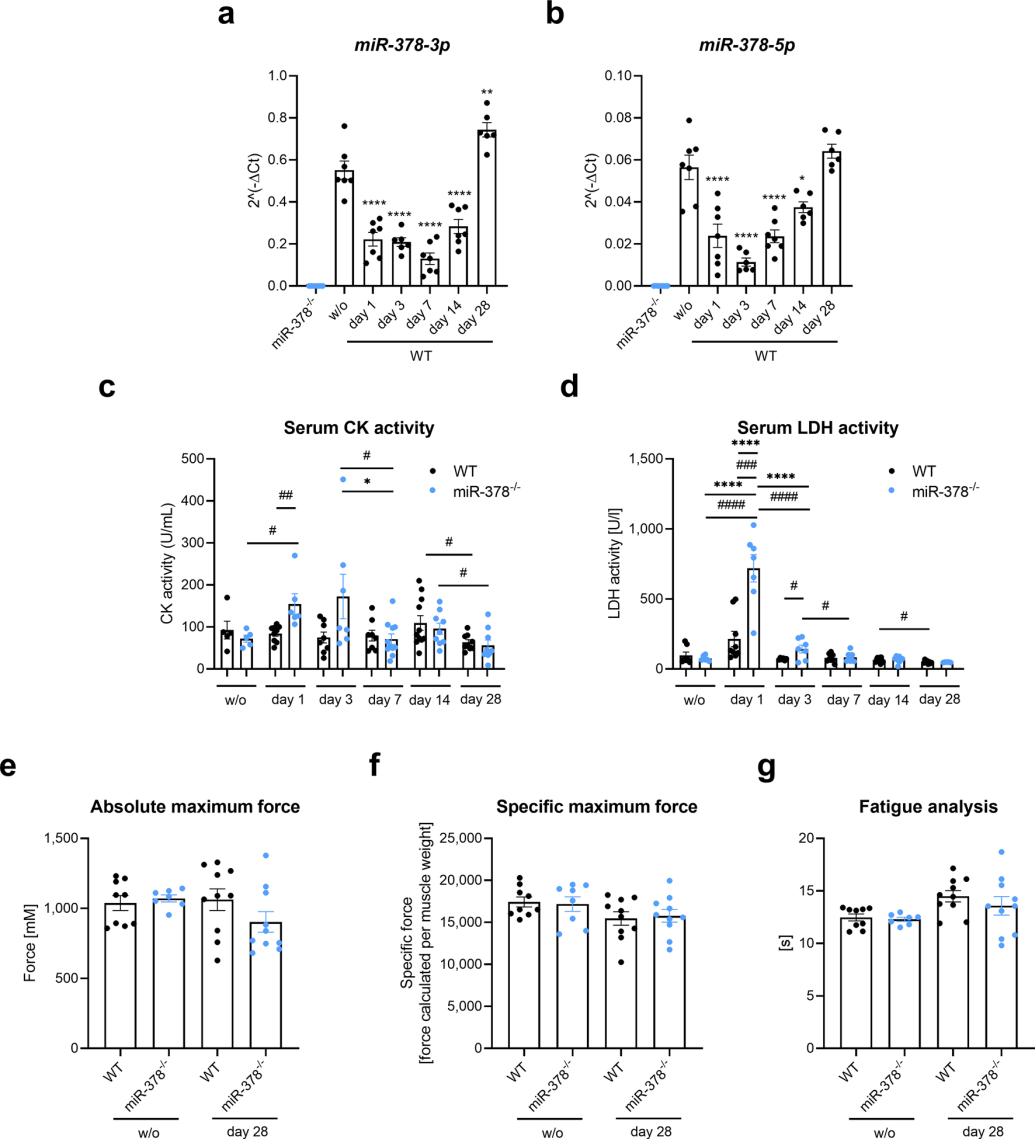
Muscle damage markers are affected by the microRNA-378 (miR-378) deletion with no influence on the contractile properties of the muscle after the injury. (**a**) miR-378-3p and (**b**) miR-378-5p expression changes during muscle regeneration. Results presented as a mean ± SEM; qRT-PCR; n = 6–7/group; *p < 0.05; **p < 0.01; ****p < 0.0001 for one-time point vs. uninjected wild-type (WT) mice (w/o) by 1-way ANOVA with Tukey’s post hoc test; (**c**) Elevated serum activity of creatine kinase (CK) during muscle regeneration; n = 5–11/group. (**d**) Increased serum lactate dehydrogenase (LDH) activity in miR-378^–/–^ animals on day 1 after injection; n = 6–11/group. Results are shown as a mean ± SEM; *p < 0.05; ****p < 0.0001 by 1-way ANOVA test with Tukey’s post hoc test; ^#^p < 0.05; ^##^p < 0.01; ^###^p < 0.001; ^####^p < 0.0001 by unpaired two-tailed Student’s *t* test. Unaffected (**e**) absolute maximum force, (**f**) specific maximum force, and (**g**) fatigue of the tibialis anterior muscle; in situ muscle contractile measurements using the Aurora system; n = 8–10/group; Results are shown as a mean ± SEM.

Initially, we analyzed the body weight (BW) of the mice to see whether the procedure of glycerol-induced injury affect the overall health of the animals. Except for a slight decrease in BW of miR-378^–/–^ animals on day 1 after injury, no alarming changes were observed (Supplementary Fig. 1a). Moreover, the expression of both miR-378 strands (3p and 5p) in the tibialis anterior (TA) muscle was significantly down-regulated from day 1 after injury, and this decreased level was maintained until day 28 (Fig. 1a,b).

To further explore the effect of miR-378 loss, we aimed to investigate muscle damage markers. A significant increase in the activity of creatine kinase (CK) and lactate dehydrogenase (LDH) after glycerol injection in miR-378^-/-^ animals on day 1 after injection was observed. Elevated CK level dropped around day 7 (Fig. 1c), whereas, for LDH, the effect was only visible on day 3 (Fig. 1d). No significant CK and LDH elevation was visible in WT mice (Fig. 1c,d).

One of the most important parts of our study was the investigation whereas potential changes caused by the lack of miR-378 during muscle regeneration could influence the functional muscle capacity. To investigate this, we measured the TA contractile properties in situ on day 28 after the injury. Our results did not show differences in the absolute (Fig. 1e) and maximal (Fig. 1f) force of the muscle. The fatigue resistance was similar in both genotypes, regardless of whether the muscle was injured or not (Fig. 1g).

### miR-378 deletion does not affect the inflammatory response

Next, when total blood cell count was conducted, we did not observe changes in the number of white blood cells (WBC) (Supplementary Fig. 1b). However, when individual populations were examined, we noticed that on day 1 after the injury, the number of monocytes was significantly elevated in both genotypes and that the increase was even higher in the knockout animals. This effect in miR-378-lacking mice was also visible on day 3 when compared to their WT counterparts (Supplementary Fig. 1c). Similarly, the number of granulocytes increased on day 1 after injury in knockout animals at the expense of lymphocytes (Supplementary Fig. 1d), while we did not observe any decline in WT equivalents (Supplementary Fig. 1e).

**Figure 2:**
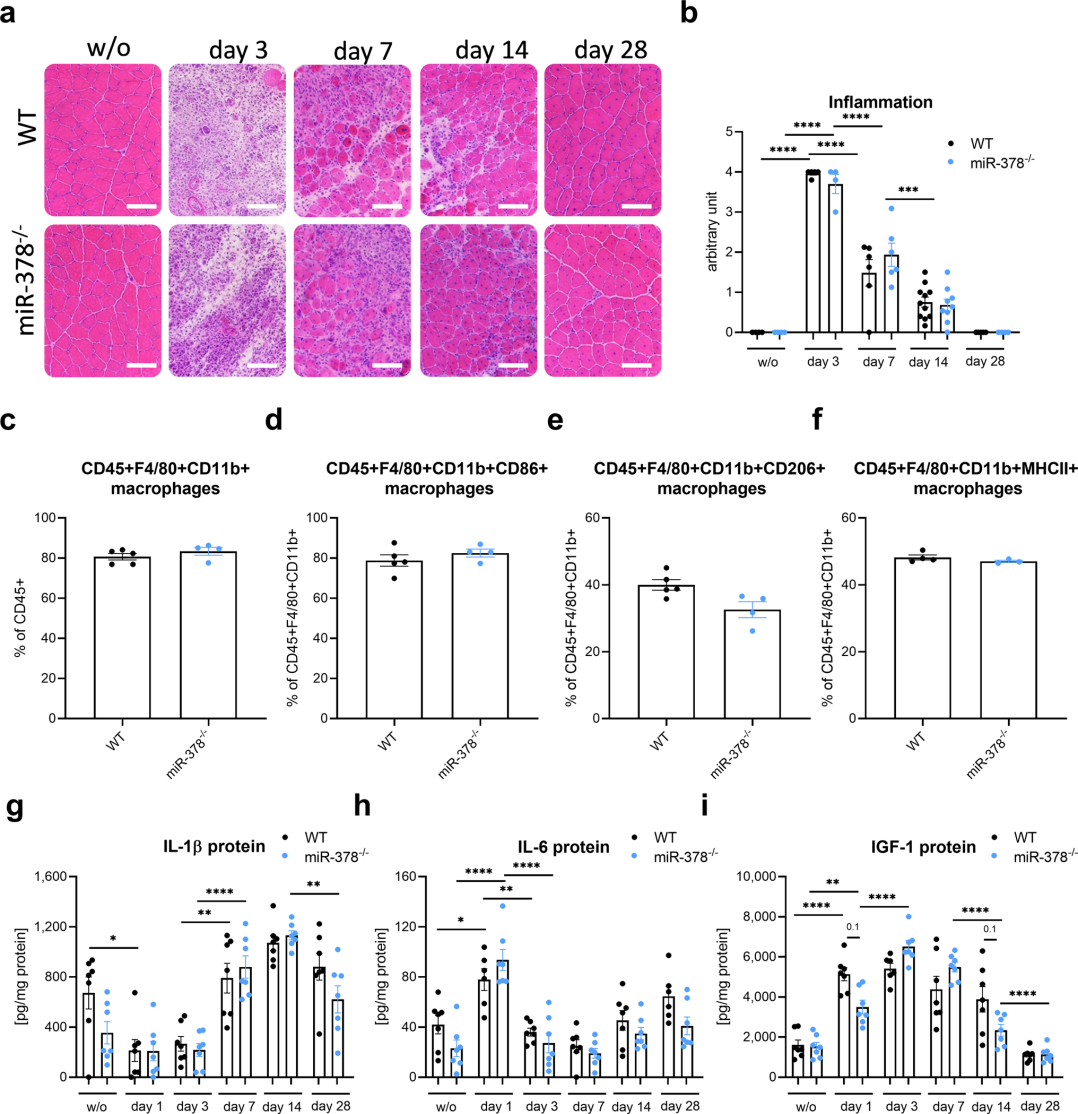
The lack of miR-378 does not affect inflammation in the glycerol-induced injury model. (**a**) Representative pictures of hematoxylin and eosin (H&E) staining with (**b**) semi-quantitative analysis of inflammation; scale bar: 100 μm; Results shown as a mean ± SEM; n = 4–11/group; Flow cytometry analysis of the (**c**) CD45 + F4/80 + CD11b + macrophages, calculated as the percentage of CD45 + cells, (**d**) CD45 + F4/80 + CD11b + MHCII + macrophages, calculated as the percentage of CD45 + F4/80 + CD11b + cells, (**e**) CD45 + F4/80 + CD11b + CD206 + macrophages calculated as the percentage of CD45 + F4/80 + CD11b + cells, (**f**), and CD45 + F4/80 + CD11b + CD86 + macrophages calculated as the percentage of CD45 + F4/80 + CD11b + cells; n = 3–4/group. Results shown as a mean ± SEM; The protein level of (**g**) Interleukin 1β (IL-1β), (**h**) Interleukin 6 (IL-6), and (**i**) Insulin-like growth factor 1 (IGF-1) in tibialis anterior muscle measured by the Luminex system; Results shown as a mean ± SEM; n = 6–7/group; *p < 0.05; **p < 0.01; ***p < 0.001; ****p < 0.0001 by 1-way ANOVA test with Tukey’s post hoc test.

Moreover, hematoxylin and eosin (H&E) staining revealed massive inflammation on day 3 that was still present to some extent up to day 14 (Fig. 2a,b). However, we did not observe significant differences between genotypes at any of the time points tested.

In accordance were also results from a flow cytometry analysis of the macrophages performed on day 3 after the injury. As the literature is not consistent in terms of which markers should be considered most appropriate to distinguish more inflammatory (M1) and more regenerative (M2) macrophages, in our study, we considered CD45^+^F4/80^+^CD11b^+^CD86^+^ and CD45^+^F4/80^+^CD11b^+^MHCII^+^ as cells with inflammatory properties and CD45^+^F4/80^+^CD11b^+^CD206^+^ as pro-regenerative, later phase-related macrophages (gating strategy of the described populations is shown in Supplementary Fig. 2). Our results showed no difference between genotypes in terms of the total percentage of macrophages and MHCII^+^, CD86^+^, and CD206^+^ populations (Fig. 2c–f).

At the same time, we measured several pro-inflammatory factors in muscle lysates at various time points after the injury. IL-4, IL-10, IL-13, and TNF-α were undetectable, whereas the level of IL-1β, IL-6, and IGF-1 did not differ between genotypes (Fig. 2g–i). Although we noticed some decreasing tendency for the IGF-1 protein, it did not reach statistical significance at any time point (Fig. 2i).

### The lack of miR-378 does not influence the classical markers of muscle regeneration

**Figure 3:**
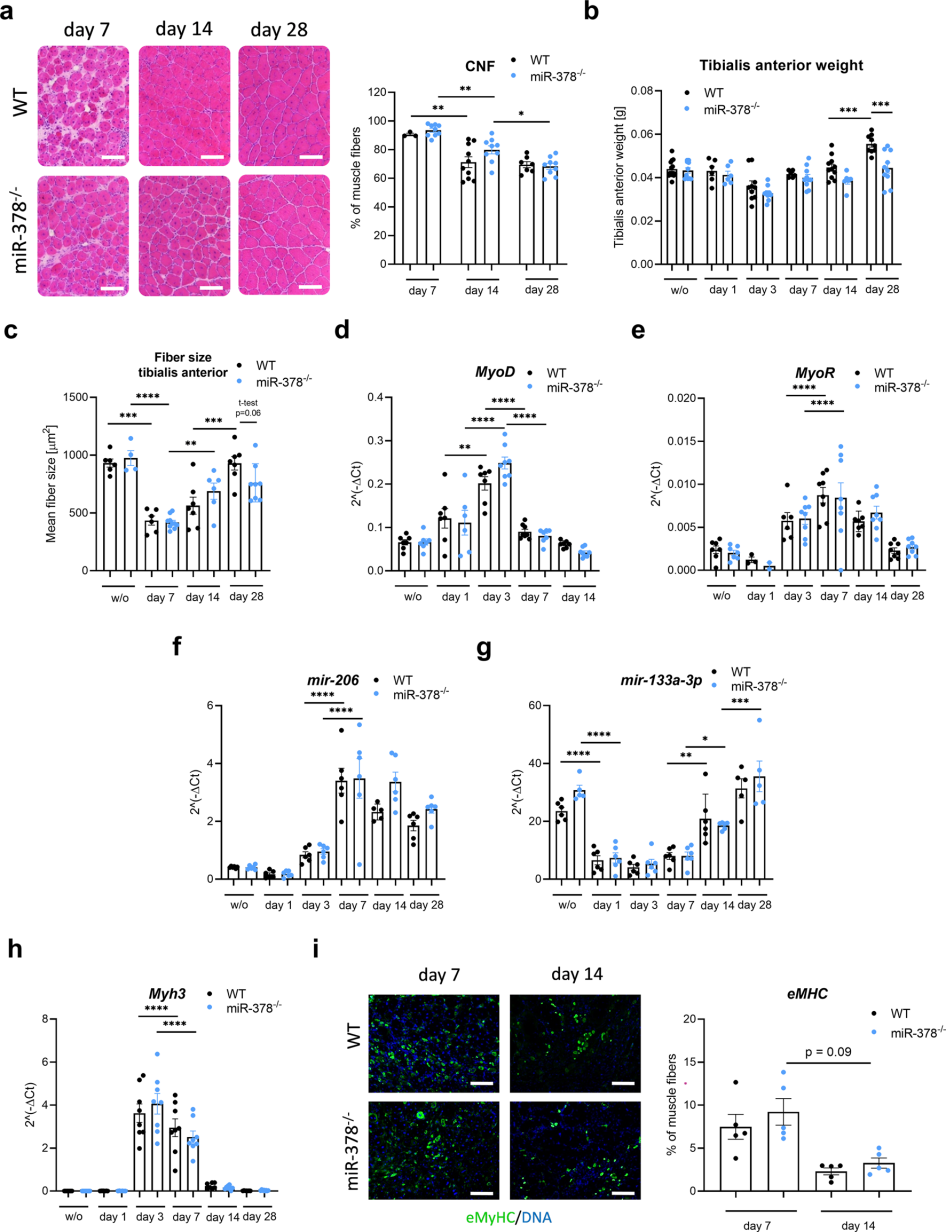
Typical markers of muscle regeneration are not affected by the lack of miR-378. (**a**) The representative photos and quantitative analysis of centrally nucleated fibers (CNF) performed based on hematoxylin and eosin (H&E) staining; scale bar: 100 μm; n = 3–10/group. (**b**) Tibialis anterior muscle mass showing reduced muscle mass in miR-378^–/–^ mice on day 28 after injury; n = 6–13/group; mean ± SEM; (**c)** Quantification of muscle fiber size based on laminin staining (not shown); n = 4–10/group; mean ± SEM. The lack of miR-378 does not influence regeneration-related factors. (**d**) myoblast determination protein 1 (*MyoD*), (**e**) myogenic repressor (MyoR), (**f**) miR-206, (**g**) miR-133a-3p, and (**h**) *Myh3* expression level; qRT-PCR; n = 5–8/group; mean ± SEM *p < 0.05 **p < 0.01; ***p < 0.001; ****p < 0.0001 by 1-way ANOVA test with Tukey’s post hoc test. (**i**) Immunofluorescent staining of embryonic myosin heavy chain (eMyHC) expression (green) with its quantification; DAPI (blue) was used to visualize DNA; scale bar: 100 μm; Results shown as a mean ± SEM; n = 5/group; 1-way ANOVA test with Tukey’s post hoc test.

In the next step, we performed a semi-quantitative evaluation of the centrally nucleated fibers (CNFs), which is one of the most commonly used methods to study regeneration^16^. H&E staining-based analysis of CNFs (usually not present in the uninjured muscle) indicated that muscles of both WT and miR-378 knockout animals were damaged and underwent regeneration. We observed a decrease in the percentage of CNFs over time but without any influence of genotype. Interestingly, on day 28 after injection, we still observed that more than 60% of the fibers are centrally nucleated (Fig. 3a). At the same time, we noticed a significant decrease in the TA weight on day 28 when miR-378 knockout animals were compared to their WT counterparts (Fig. 3b).

The analysis of muscle fibers during regeneration showed that the mean size of the fibers on day 28 is similar to that observed in undamaged muscles for both genotypes (Fig. 3c). Moreover, when compared to the uninjured muscle (Supplementary Fig. 3a), the muscle fibers on days 7 and 14 were much smaller (Supplementary Fig. 3b,c). On day 28, we can still observe a substantial number of small fibers (Supplementary Fig. 3d).

Furthermore, miR-378 did not affect the mRNA level of one of the main factors regulating muscle differentiation, *MyoD* (Fig. 3d) and *MyoR* (Fig. 3e) as well as muscle-specific miRNAs, miR-206 and miR-133a-3p (Fig. 3f,g) or one of the most frequently utilized markers of muscle regeneration, eMyHC, as shown on mRNA expression (*Myh3)* (Fig. 3h) and protein staining and quantification (Fig. 3i).

### The abundance of mSCs is elevated in miR-378-lacking animals

**Figure 4:**
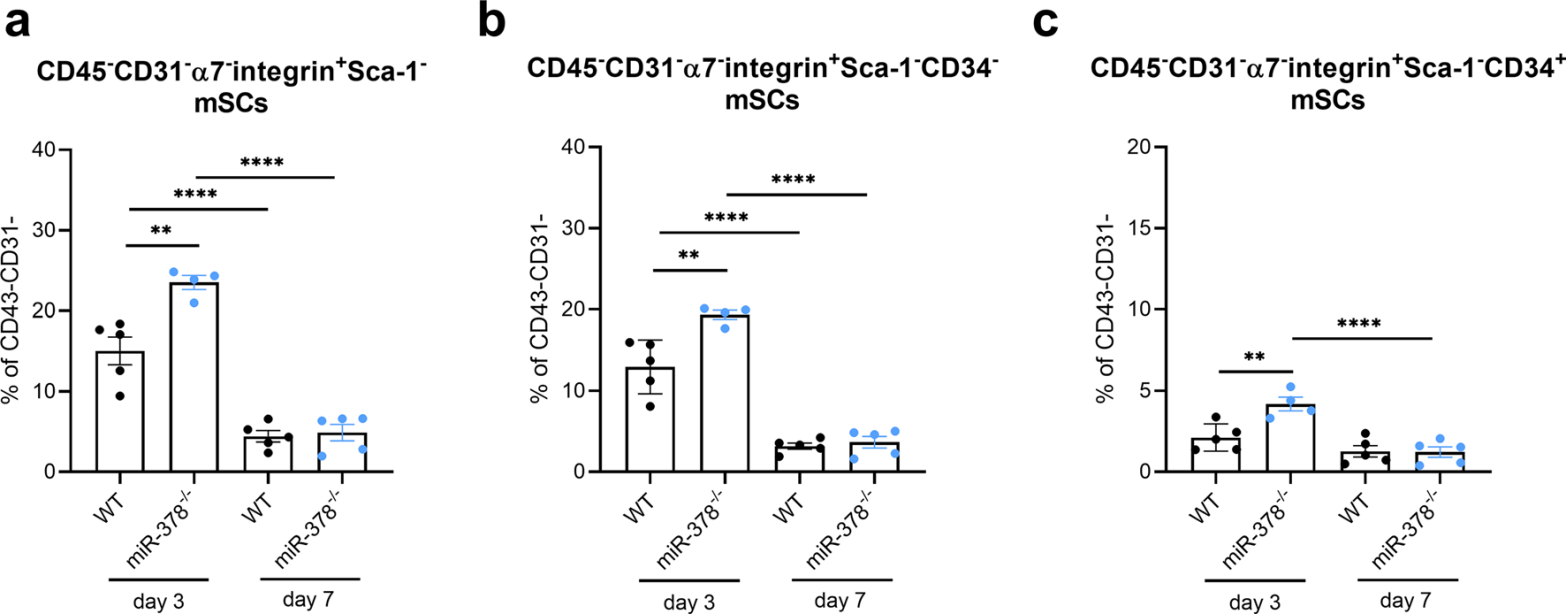
miR-378 depletion leads to the increased percentage of muscle satellite cells (mSCs) on day 3 after injury. The analysis of (**a**) total, (**b**) activated (CD34^–^), and (**c**) quiescent (CD34^+^) mSCs (CD45^–^CD31^–^Sca1–α7i^+^) in tibialis anterior muscle on days 3 and 7 after glycerol injection; flow cytometry analysis; n = 4–5/group; mean ± SEM; **p < 0.01; ***p < 0.001; ****p < 0.0001 by 1-way ANOVA test with Tukey’s post hoc test.

Muscle regeneration would be impossible without mSCs. Hence, we estimated the percentage of mSCs on days 3 and 7 after glycerol injection using flow cytometry with an appropriate gating strategy (Supplementary Fig. 4a,b). Our data revealed a significantly higher percentage of total, activated, and quiescent mSCs (Fig. 4a–c, respectively) on day 3 when miR-378-lacking mice were compared with WT animals.

### Deletion of miR-378 does not affect fibrosis

**Figure 5:**
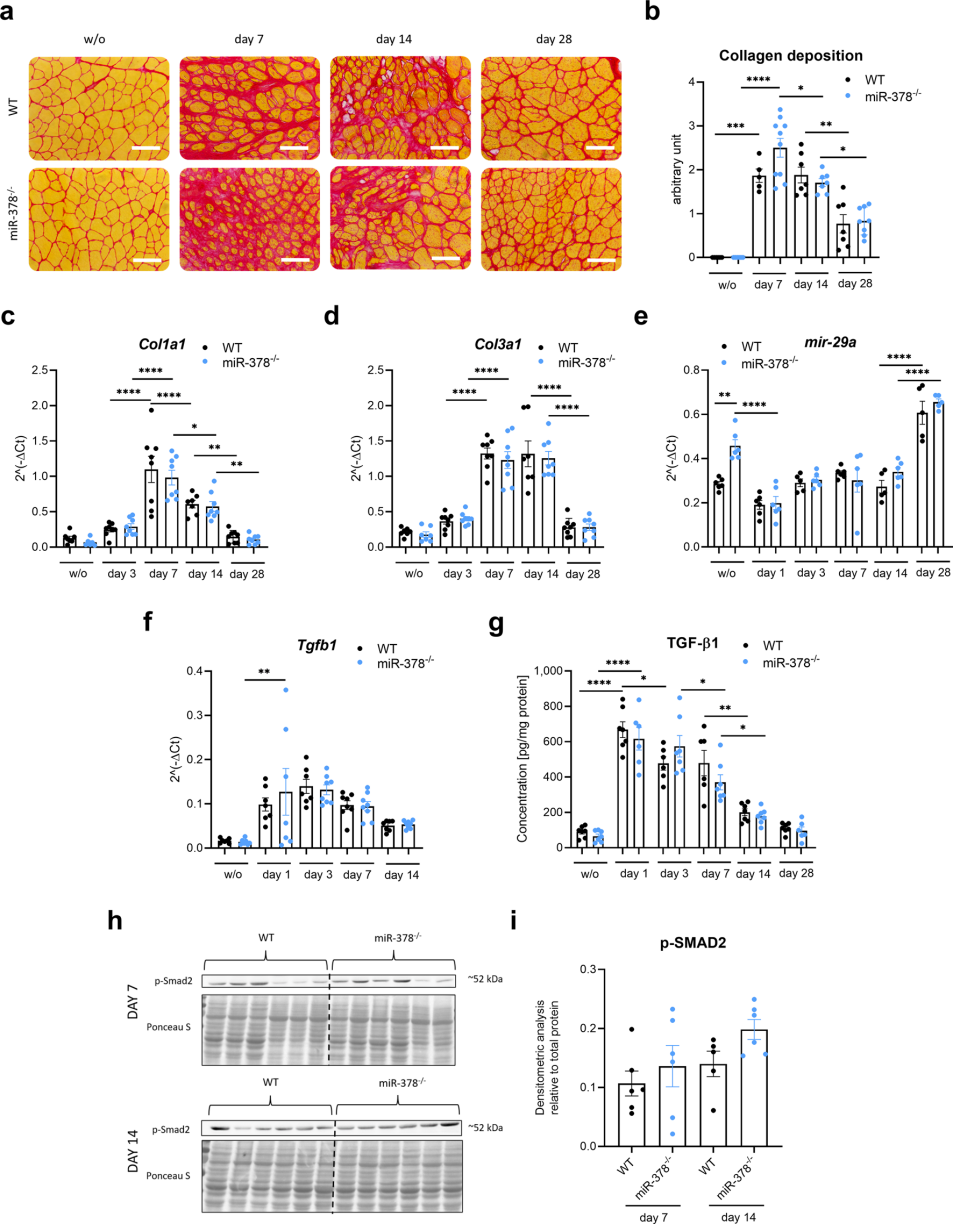
Collagen deposition is not affected by the lack of miR-378 in the glycerol-induced injury model. (**a**) Representative photos of Sirius red staining with (**b**) semi-quantitative analysis of collagen deposition; microscopic assessment; scale bar: 100 μm; (**c**) *Col1a1,* (**d**) *Col3a1*, and (**e**) miR-29a level; qRT-PCR; n = 5–8/group; mean ± SEM; n = 5–10/group; (**f**) Expression of transcript level: *Tgfb1;* qPCR; n = 7–8/group; mean ± SEM. (**g**) Protein level of TGF-β1; ELISA; n = 6–7/group; mean ± SEM. The protein level of phosphorylated SMAD Family Member 2 (SMAD2) protein demonstrated on (**h**) Western blot and after (**i**) densitometric analysis; Ponceau S staining was used as loading control; n = 5–6/group; *p < 0.05 **p < 0.01; ****p < 0.0001 by 1-way ANOVA test with Tukey’s post hoc test.

One of the main characteristics of glycerol-induced injury is the appearance of fibrous tissue accumulation in the later stages of the regeneration process. Our initial analysis of the Sirius red staining (Fig. 5a) showed a substantial accumulation of fibrous tissue on days 7 and 14, which was still evident on day 28 after the injury (Fig. 5a,b). No differences between genotypes were observed.

Similarly to the estimation of collagen deposition (Fig. 5a,b), we did not observe any difference in the mRNA level of *Col1a1* and *Col3a1* between genotypes (Fig. 5c,d). However, even though no changes were noticed during regeneration, the basal expression of anti-fibrotic miR-29a^17^ was significantly elevated in miR-378 knockout animals (Fig. 5e).

One of the most important factors regulating muscle fibrosis is TGF-β1^18^. We analyzed both mRNA and protein levels of this growth factor and showed strong induction of its expression during muscle regeneration without significant differences between genotypes (Fig. 5f,g). Additional Western blot analysis of SMAD2 phosphorylation (TGF-β1 downstream signaling pathway) with densitometric analysis confirmed no effect of miR-378 on the TGF-β1 pathway (Fig. 5h,i).

### miR-378 deletion does not affect adipogenesis

**Figure 6:**
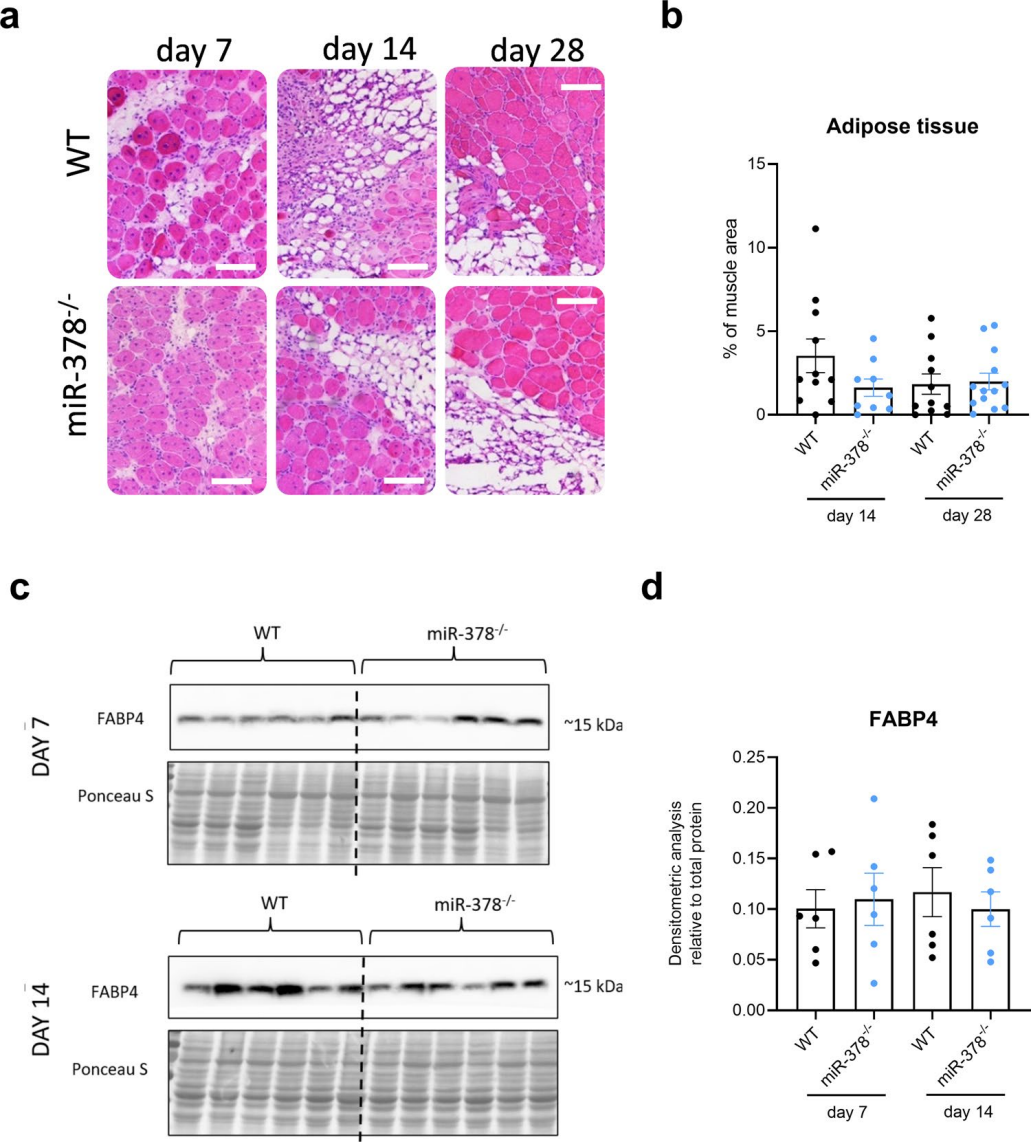
Lack of miR-378 does not affect adipose tissue accumulation on days 7, 14 and 28 during regeneration. (**a**) Representative photos of H&E staining with (**b**) semiquantitative analysis of adipose tissue accumulation; microscopic assessment; scale bar: 100 μm; n = 9–13/group. The protein level of Fatty acid-binding protein 4 (FABP4) demonstrated on (**c**) Western blot and after (**d**) densitometric analysis; Ponceau S staining was used as loading control; n = 6/group.

The glycerol-induced injury is one of the best models for studying adipogenesis during muscle regeneration^19^. In our experiments, we were able to detect the accumulation of adipose tissue starting from day 7 after damage. We did not observe significant changes between genotypes on day 14 or day 28 (Fig. 6a,b). Moreover, the protein level of FABP4, highly expressed in adipocytes, was the same in animals of both genotypes (Fig. 6c,d).

### FAPs isolated from muscles of miR-378^–/–^ animals differentiate more preferentially to adipocytes in culture

**Figure 7:**
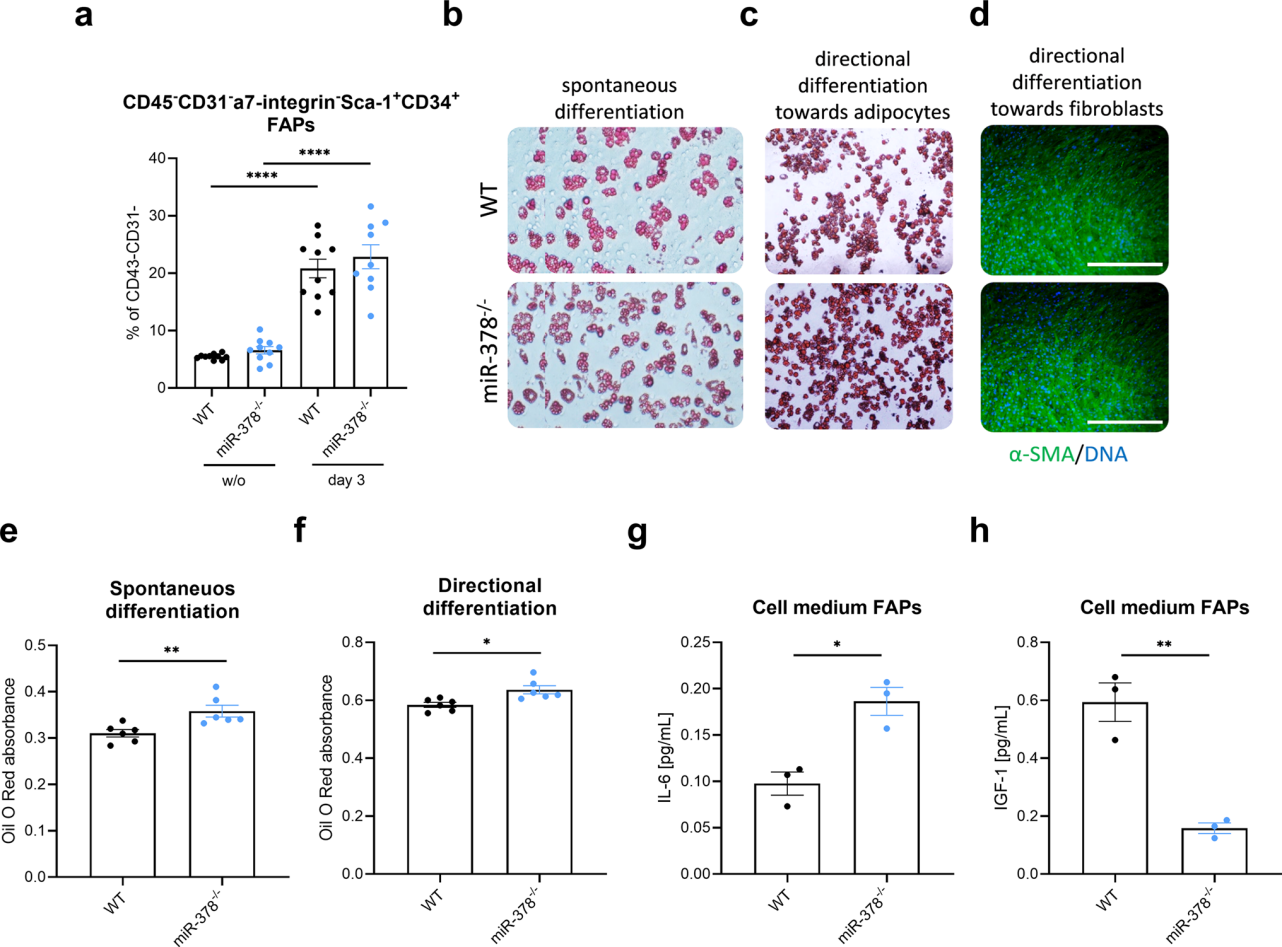
miR-378^–/–^ fibro-adipogenic progenitors (FAPs) differentiate more preferentially into the adipocytes when compared to the WT cells. (**a**) Unaffected by the lack of miR-378 abundance of FAPs in mice without injury and on day 3 after glycerol injection, identified as CD45^–^CD31^–^Sca1^+^α7i^–^CD34^+^ cells; flow cytometry analysis calculated as the percentage of CD45^-^CD31^–^ cells; n = 9–10/group. Data are presented as mean ± SEM; ****p < 0.0001 by 1-way ANOVA test with Tukey’s post hoc test. Representative photos of (**b**) spontaneous differentiation towards adipocytes and directional differentiation towards (**c**) adipocytes and (**d**) fibroblasts based on the Oil Red O (for adipocytes (**b**,**c**)) and α-smooth muscle actin (α-SMA) (for fibroblasts (**d**)) staining; DAPI was used for DNA staining; scale bar: 500 μm. Absorbance measurement of Oil Red O stain extracted from stained adipocytes after (**e**) spontaneous and (**f**) directional differentiation towards adipocytes; mean ± SEM; n = 6. (**g**) IL-6 and (**h**) IGF-1 protein level in medium from FAPs culture; Luminex; mean ± SEM; n = 3; *p < 0.05 **p < 0.01 by unpaired two-tailed Student’s *t* test.

Both fibrosis and adipogenesis are related to the function of FAPs with their unique ability to differentiate into ECM-producing fibroblasts and adipocytes^20^. On day 3 after glycerol injection, utilizing proper gating strategy (Supplementary Fig. 4a,c), we observed a significant increase in the percentage of FAPs in muscles. However, no difference was noted between genotypes (Fig. 7a). As the involvement of FAPs during muscle regeneration relies on their strictly regulated differentiation potential we decided to perform additional experiments using cells isolated from injured muscles.

Therefore, we sorted WT and miR-378-lacking FAPs on day 3 after injury and performed two types of experiments: spontaneous differentiation, where cells were left in a culture medium without additional stimuli, and directional differentiation where cells were cultured in adipogenic or fibrotic medium to activate specified pathways. Using Oil Red O staining to label adipocytes, we demonstrated that, independently whether it was spontaneous (Fig. 7b) or directional (Fig. 7c) differentiation, FAPs without miR-378 created adipocytes more preferentially than WT cells while maintaining their fibrogenic potential (Fig. 7d). The higher adipogenic potential was also additionally confirmed when Oil Red O was extracted from cells (Fig. 7e,f). In the next step, while analyzing collected from cells media, we observed that FAPs without miR-378 produce more IL-6 and significantly less IGF-1 than their WT counterparts (Fig. 7g,h).

### FAPs transcriptome is not strongly affected by the lack of miR-378

**Figure 8:**
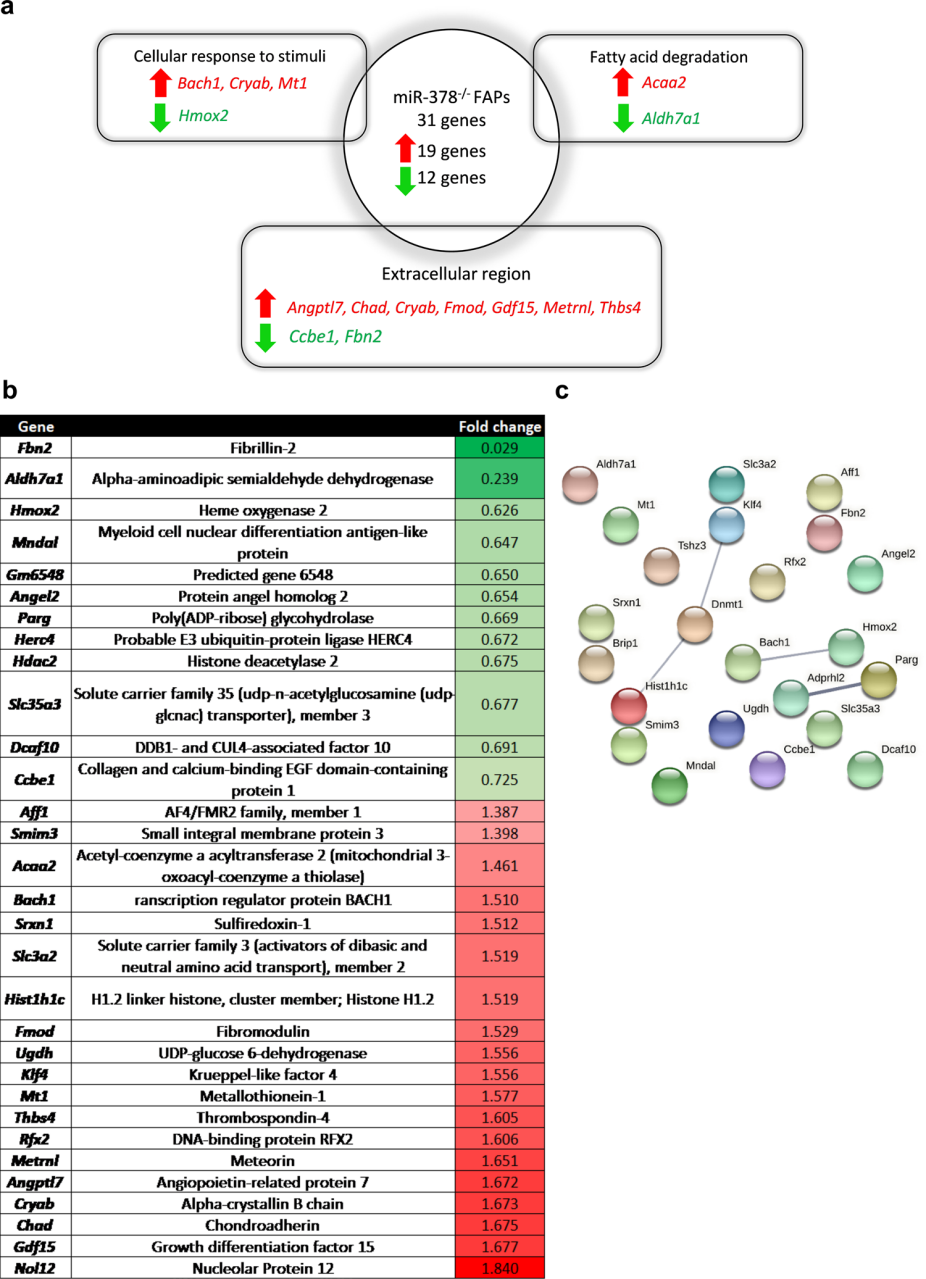
Deletion of miR-378 does not significantly affect the transcriptome of FAPs. (**a**) Basic analysis using the Database for Annotation, Visualization and Integrated Discovery (DAVID) database showing examples of processes that were significantly enriched based on our transcriptomic analysis. Red arrows show how many genes were upregulated in miR-378^–/–^ mice. Green arrows show how many genes were downregulated in miR-378^–/–^ mice. p_adj_ value < 0.05; (**b**) List of all significantly upregulated and downregulated genes with (**c**) lack of apparent connection between them (prepared using Search Tool for the Retrieval of Interacting Genes/Proteins (STRING) database). The thickness of the line reflects the confidence of the protein–protein interaction.

To better understand observed results, FAPs from WT and miR-378 knockout mice were sorted for RNA sequencing on day 3 after injury. Transcriptional analysis revealed only 31 differentially expressed genes with a p_adj_ value < 0.05 (Fig. 8a).

Among them, 19 genes were up-regulated and 12 down-regulated in FAPs isolated from miR-378^–/–^ mice. Basic functional annotation analysis using the Database for Annotation, Visualization, and Integrated Discovery (DAVID) revealed that two genes were connected to the fatty acid degradation pathway and four to the cellular response to stimuli. Furthermore, 9 of 31 genes were related to the extracellular region, suggesting their role in ECM function (Fig. 8a). When all identified genes (Fig. 8a,b) were analyzed for any potential interactions using the STRING database, nothing significant was found (Fig. 8c).

When we decided to expand our search and changed the p_adj_ value to < 0.1 (p_value_ to 0.0008), we observed significant changes in genes from the TGF-β signaling pathway and the ECM-receptor interaction. In addition, an even higher number of genes related to cellular stress were identified (Supplementary Fig. 5). All significantly changed genes (p_adj_ value to < 0.1) are listed in Supplementary Table 1. Importantly, in order to draw the final conclusions based on those results, thorough verification of the transcriptomic data would be required.

## Discussion

Muscle regeneration is a highly organized process that requires the strictly controlled cooperation of several types of cells, including inflammatory cells, mSCs, fibroblasts, FAPs, and many others^21^. Deviations in the timing and/or strength of particular interactions could lead to substantial alterations in the function of the regenerative machinery and, as a result, insufficient regeneration, accumulation of fibrous tissue, and weakening of the muscle. All of the above are classic symptoms of Duchenne muscular dystrophy (DMD) progression^22,23^.

Despite many years of research, there is still no effective therapy for this disease. We have previously demonstrated the importance of miR-378 in the mouse model of DMD^9,11^, and in the present study, we investigated the effect of its deletion in the glycerol-induced acute injury model in WT mice.

miR-378 is a small non-coding RNA with a role described in lipid and glucose metabolism, inflammation, and muscle regeneration^24^. Our previous results showed that miR-378 alters carbohydrate and lipid metabolism in *mdx* mouse DMD model^11^. Moreover, the global deletion of miR-378 in the dystrophic mice resulted in better physical performance and improved muscle force, together with a decrease in inflammation, the abundance of regenerating fibers, FAPs, and fibrosis^9^. To shed more light on the possible interaction between miR-378 and FAPs and to better control the following phases and different processes contributing to muscle regeneration, in the present study we decided to use the acute glycerol-induced injury model. It is a well-controlled and repeatable acute model of muscle injury and, to the best of our knowledge, its use was never connected with the investigation of miR-378. Most importantly, due to the course of degeneration and the appearance of connective and fatty tissue, it is suggested to be a suitable model for studying the pathophysiology of DMD that may allow us to at least partially translate our results into the dystrophic phenotype^12,25^.

In our experiments, we noticed an initial drop in the expression of both mir-378 strands (3p and 5p), which lasts for several days after the injury. Interestingly, previously, we observed a very similar pattern of expression of miR-378 in mice subjected to femoral artery ligation (FAL)^8^. Importantly, we have also demonstrated significantly lower expression of miR-378 in the gastrocnemius of dystrophic animals^9^. The variance in the expression pattern of miR-378 could be explained by the release of miR-378 from damaged myofibers or the active secretion of miR-378 exhibiting paracrine function. It should be noted that, concomitantly with the decrease in muscles, an increased level of miR-378 detected in plasma/serum, may serve as a potential marker of damaged muscles^9,10^.

We have demonstrated that glycerol injection led to muscle injury, as we found substantial infiltration of inflammatory cells with a peak on day 3 after the injury, changes in the abundance of various subpopulations of WBC in the blood of affected animals, including a substantial increase in the percentage of monocytes and granulocytes in peripheral blood, and elevated CK and LDH activity. However, miR-378 effect on all the above processes was temporal and observe only in the early stages after injury, indicating lack of its crucial role on the overall outcome of inflammation-driven regeneration. Interestingly, the increase in the CK and LDH activity was not as potent as we would have expected, especially in WT animals. It should be emphasized that enzymes such as CK and LDH are released into the circulation immediately after injury, but they have a short half-time and are removed very quickly by absorption by the liver and kidney^26,27^. The lack of effect could potentially be explained by the use of a different injury model; however, a potent elevation was demonstrated by Kawai et al*.* 24 h after glycerol administration^25^. Another explanation could be related to the fact that the tibialis anterior muscles, which were injected with glycerol, are relatively small, and therefore, we were unable to detect potential effects. Nevertheless, despite observed changes, there is no effect on the overall outcome of muscle regeneration at the end of the experiment.

As we observed extensive damage to the muscle, we aimed to investigate whether the lack of miR-378 could affect the outcome of regeneration, which means restoration of muscle function. We showed that the absolute and specific maximum force of the muscle and the fatigue resistance were similar between the uninjured muscle and on day 28 after the injury, regardless of the genotype of the mice. These results do not confirm our data from *mdx*/miR-378^–/–^ mice, where the lack of miR-378 resulted in improved muscle strength in dystrophic animals^9^. Further histological analysis revealed a substantial increase in the number of the CNF, confirming the preceding damage to the tissue. When the percentage of CNF was independently analyzed, at various time points after glycerol administration, we noticed that on day 28, more than 60% of all fibers are still centrally nucleated, which, despite apparent function restoration, supports the hypothesis of delayed and incomplete muscle regeneration in the glycerol-induced injury model^12^.

Our results indicating that miR-378 deletion does not affect muscle regeneration are further supported by gene and microRNA expression analysis. In our hands, similarly to the published results^28^, the expression of *MyoD* was induced and continued during the proliferative phase of mSCs and persisted until the first differentiation phase. However, we have not observed the effect of miR-378 on MyoR, an inhibitor of MyoD, although this factor was suggested as a target of miR-378^29^. Changes in miR-206 and miR-133a, the so-called myomirs^30,31^, also did not indicate any genotype-related alterations. Nevertheless, when we investigated the abundance of mSCs, the key player in muscle regeneration^32^, we noticed that on day 3 post-injury, there is a higher percentage of these *bona fide* muscle stem cells in animals lacking miR-378, including both activated and quiescent ones. It could suggest a more potent activation of these cells. Interestingly, in our work on the role of miR-378 in dystrophic animals, we noted a lower number of activated mSCs in dystrophic mice without miR-378, indicating a different regulation of this process in the tested models^9^. It seems that the role of miR-378 is more related to the early stages of injury, as most of the observed differences between genotypes are present at that time point. As we found significant increase in the IGF-1 protein level in miR-378-lacking animals between day 1 and 3, proliferation and activation studies would be reasonable step forward approach. It was shown in the literature that elevated IGF-1 levels are required for mSC and myoblast proliferation and post-injury regeneration^33^. Interestingly, miR-378 can functionally inhibit muscle cell differentiation by targeting *Igf1r*, which was demonstrated to be a direct target of miR-378^34^. Although the changes in the abundance of mSCs are interesting, we would like to acknowledge, that we did not observe their influence on later and final stages of regeneration.

Disruption of regeneration leads to the excessive accumulation of the extracellular matrix (ECM) components and substitution of muscles with fibroadipose tissue. The effect of miR-378 on fibrosis appears to be complex and tissue-dependent^35–37^. Mahdy et al*.*^13^ showed that collagen deposition starts on day 4 after glycerol-induced injury and is still prominently present in the late stage of regeneration (day 14 after injury). We demonstrated time-dependent accumulation of fibrous tissue and even 28 days after the injury, we still observed signs of fibrosis present in muscle tissue. However, since no changes were found in functional evaluation, it may not be enough to alter muscle function through e.g. creating a barrier between muscle and capillaries^38,39^. Additionally, we did not notice genotype-related changes in the expression of *Col1a1* and *Col3a1* or miR-29a. The role of miR-29a in fibrosis was clearly demonstrated in various conditions. Overexpression of miR-29a in muscle cells decreased collagen levels^40^ and its downregulation leads to increased ECM protein expression^41^. Additionally, TGF-β signaling was shown to be responsible for decreased miR-29a expression levels during the course of collagen deposition^42^. Nevertheless, despite miR-29a upregulation in uninjured miR-378 knockout animals, we did not notice any effect of miR-378 deletion on either miR-29a levels during regeneration or TGF-β1 mRNA and protein levels. Moreover, no changes in one of the TGF-β1-related signaling pathways, SMAD2 phosphorylation^43^, were noticed. In general, we did not observe any effect of miR-378 deletion on muscle fibrosis, which is contrary to our findings in *mdx* mice without miR-378. Once again, it suggests different mechanisms that regulate fibrous tissue accumulation in both models.

One of the advantages of using a glycerol-induced injury model is the ability to analyze the deposition of adipose tissue in the muscle during regeneration. Injection of glycerol influences the cytokine balance in tissue and alters signals sent to mSCs, myoblasts, and adipocyte progenitors, resulting in increased adipogenesis^44^. In our study, we did not find any effect of miR-378 deletion on adipose tissue content.

Both fibrosis and accumulation of adipose tissue are connected to the function of FAPs. The name of these cells is derived from their potent ability to differentiate into fibrogenic (transcription factor 4 (Tcf4^+^), α-SMA^+^) and adipogenic lineage that was presented both in vitro and in vivo^15,20,45^*.* Due to their dual potential, the possible influence on DMD is highly studied. Dysregulation in inflammation-regulated FAP dynamics is one of the factors affecting pathogenic outcomes^46^. In *mdx*/miR-378^–/–^ mice, the potently decreased abundance of FAPs was found^9^. In the current research, we did not observe any differences in the percentage of FAPs, neither in mice non-injected with glycerol nor on day 3 after the injury (peak in the FAPs number suggested in the literature^46^). Moreover, the additional, transcriptomic analysis of the cells isolated from injured muscle (day 3), did not demonstrate many genes differentially expressed between both genotypes. The ones that did significantly change suggest targets for the future studies verifying the transcriptome data. However, we found that miR-378-lacking FAPs sorted from the muscle on day 3 after injury more preferentially created adipocytes and produced significantly more IL-6 and less IGF-1 (playing an important role in the activation and proliferation of mSCs^47,48^) than FAPs isolated from WT mice. Whether it influences the interaction between mSCs and FAPs is still an open question, especially as we do not observe any significant difference in fibrosis and adipogenesis between genotypes. We believe that observed changes might be related to the sensitivity of the FAPs to the environmental factors and inflammatory (both pro- and anti-) conditions in the muscle, which are not present in the in vitro culture, might have a much stronger effect on FAPs biology. Though, it requires further studies.

## Conclusion

In conclusion, our study revealed that miR-378 does not play a crucial role in muscle regeneration in response to glycerol-induced muscle injury in WT animals. However, it can be considered a factor influencing the differentiation and secretion profile of FAPs. These important aspects, discovered for the first time, warrant further research, especially in the context of the interaction of FAPs with mSC.

## Material and methods

### Mouse models

All experiments were performed following the national and European legislation on the in vivo research and in accordance with the Directive of the European Parliament and of the Council 2010/63/EU of 22 September 2010 on the protection of animals used for scientific purposes and ARRIVE guidelines. All experimental procedures were approved by the 2nd Institutional Animal Care and Use Committee (IACUC) in Kraków, Poland (approval numbers: 299/2019 and 314/2020). All experiments were carried out on 6-week-old, wild-type (WT) and global miR-378 knockout (miR-378^–/–^) male mice (C57BL/6 background). miR-378^–/–^ mice (129SvEv/C57BL/6 background) were kindly gifted by Prof. Eric Olson (Department of Molecular Biology, University of Texas Southwestern Medical Center, Dallas, Texas, USA)^49^. In our animal facility, mice were backcrossed with C57BL/6 mice for 10 generations to obtain mice on a clear C57BL/6 background. The animals were housed in specific pathogen-free (SPF) conditions with water and food available ad libitum, and under controlled temperature (approximately 23 °C), humidity (around 55 ± 10%), and 14 h (h)/10 h light/dark cycles.

### Glycerol-induced injury model

Mice were injected intramuscularly (tibialis anterior (TA) muscle on both legs of the mice) with 50 µl of 50% glycerol solution (Sigma-Aldrich) diluted in distilled water (dH_2_O) according to the published protocol^50^. On the day of injection and the following day, mice received subcutaneously analgesic buprenorphine (0.1 mg/kg body weight) (Polfa Warszawa SA). The material was collected at the appropriate time points: 1, 3, 7, 14, and 28 days after injection and from uninjured animals (labeled as w/o).

### Muscle contractile properties

The specific maximum force of the TA muscle was evaluated in situ 28 days after the glycerol injection using Aurora 1300A: 3-in-1 Whole Animal System (Aurora Scientific) and based on an already established protocol^9^. The absolute maximal force was determined after stimulation with the trains of stimuli with increasing frequencies from 50 to 150 Hz. The specific maximal force was calculated by dividing the absolute muscle force by the TA muscle weight. The fatigue experiment was conducted by applying continuous tetanic stimulation (30 s stimulation at 50 Hz). The muscle fatigue was then determined as the time after which a decrease in muscle force was achieved by 50% of the base value.

### Blood cell count

Blood was extracted directly from the *vena cava* and collected in ethylenediaminetetraacetic acid (EDTA)-coated tubes. The total number of white blood cells (WBCs) and the percentage of granulocytes, monocytes, and lymphocytes among WBCs were then analyzed using scil Vet abc instrument (Horiba ABX).

### Histological and immunofluorescent analyses of the muscles

#### Frozen tissue sections preparation

Half of the TA muscles from each time point (days 1, 3, 7, 14, and 28) were dissected from the euthanized mice and immediately snap-frozen in an optimal cutting temperature compound (OCT, Leica) in liquid nitrogen-cooled isopentane, and stored at − 80 °C. The 10 µm frozen tissue sections were used for all histological and immunofluorescence stainings.

### Hematoxylin and eosin (H&E) and Sirius red stainings

Before staining, frozen sections were fixed with 4% buffered formalin (pH 7.4) (Sigma-Aldrich).

For **H&E staining** (assessing inflammation and regeneration), sections were incubated with hematoxylin solution (Sigma-Aldrich) for 12 min, washed with running tap water for ~ 15 min, and stained with 0.1% eosin (Sigma-Aldrich) for 1.5 min. For **Sirius red staining** (evaluating fibrosis, i.e. collagen deposition), frozen sections were incubated with hematoxylin solution (Sigma-Aldrich) for 10 min, washed with running tap water for ~ 10 min, stained in Picro-Sirius red solution (1% Sirius red (Sigma-Aldrich) in the saturated aqueous solution of picric acid (Sigma-Aldrich) for 60 min, and washed twice with acidified water (0.5% acetic acid (Avantor Performance Materials Poland SA) in dH_2_O). Finally, slides subjected to H&E and Sirius red staining were dehydrated by increasing the concentration of ethanol (50%, 70%, 96%, 99.9%; Avantor Performance Materials Poland S.A.), cleared in xylene (Sigma-Aldrich), and mounted using Histofluid (Chemilab).

Pictures of slides subjected to H&E and Sirius red staining were taken using the Leica DMi8 microscope with the CMOS Leica MC170 HD camera or the Nikon Eclipse Ti microscope. The scoring (arbitrary units: 0–4) of inflammation, regeneration (based on the abundance of centrally nucleated fibers; CNF), and fibrosis was performed by a blinded experimenter, as previously described^9,51^. For the analyses, 10–15 pictures per tissue section were taken randomly.

### Immunofluorescent (IF) stainings

Embryonic myosin heavy chain (**eMyHC)** staining was performed as described previously^9,52^. Images were acquired using a Leica DMi8 microscope with Leica DFC7000 GT fluorescent camera. The analysis was performed by the experimenter blinded to the mouse genotype and/or treatment. The cross-sectional area (CSA) and the mean fiber area were determined by semiautomatic muscle analysis using histological segmentation (SMASH)^53^ based on the immunofluorescent staining of laminin^54^. The percentage of eMyHC-positive fibers was counted in the whole scan of the tissue within all injured sites and calculated in relation to the total number of myofibers in the region of interest.

### Creatine kinase (CK) and lactate dehydrogenase (LDH) activity in serum

Serum was obtained by blood collection from the *vena cava* to Eppendorf tubes. The activity of CK and LDH was measured using the diagnostic Liquick Cor-CK and Liquick Cor-LDH kit, respectively (Cormay), following the manufacturer’s instructions. The absorbance values were then converted to CK and LDH (U/l).

### Flow cytometry analysis of macrophages, FAPs, and mSCs

TA muscles were excised on day 3 after glycerol injection and the abundance of the cells was analyzed using flow cytometry. Preparation of the samples was performed in accordance with the protocols described previously^9,51^.

To analyze macrophages, FAPs, and mSCs, cells were first incubated with rat anti-mouse CD16/CD32 antibody to block binding to the Fc receptor, and then with the appropriate antibodies listed in Table 1.

**Table 1:** Antibodies used for FACS analysis of macrophages, fibro-adipogenic progenitors (FAPs), and muscle satellite cells (mSCs).

Staining	Antibody	Company	Clone/catalog number
All	rat anti-mouse CD16/CD32	eBioscience	93
Macrophages	rat anti-mouse CD45-FITC	BD Biosciences	30-F11
	rat anti-mouse CD11b-Alexa Fluor 700	eBioscience	M1/70
	rat anti-mouse F4/80-APC	eBioscience	BM8
	rat anti-mouse MHCII-PE-Cy7	BD Biosciences	M5/114.15.2
	rat anti-mouse CD206-PerCP/Cy5.5	BioLegend	C068C2
	rat anti-mouse CD86-PE	eBioscience	GL1
FAPs and mSCs	rat anti-mouse CD45-FITC	BD Biosciences	30-F11
	rat anti-mouse CD31-FITC	BD Pharmingen	MEC 13.3
	rat anti-mouse Ly6A/E-PE-Cy7 (Sca-1)	eBioscience	D7
	rat anti-mouse α7-integrin-APC	R&D Systems	#334908
	rat anti-mouse CD34-PE	BD Pharmingen	RAM34

Data were acquired using a Fortessa flow cytometer (BD Biosciences) and analyzed in FACS Diva software (BD Biosciences). Gates were set based on the appropriate Fluorescence Minus One (FMO) controls. Fluorescence compensation for each fluorochrome was performed before every new experiment. Results are presented as a percentage of CD45^+^ cells, CD45^+^ F4/80^+^ CD11b^+^ or CD45^-^CD31^–^ cells. Different cell populations were evaluated based on the appropriate gating strategy (Supplementary Figs. 2, 4).

### Fluorescence-activated cell sorting (FACS) of FAPs

To isolate FAPs from WT and miR-378^–/–^ on day 3 after glycerol-induced muscle injury, skeletal muscles were prepared similarly as for flow cytometry analysis. 10,000 cells/well of CD45^-^CD31^–^α7-integrin^-^Sca-1^+^CD140a^+^ FAPs were sorted for cell culture experiments and transcriptome sequencing.

### FAP culture and differentiation

10 000 per well of sorted FAPs were seeded on a non-treated culture plate in the growth medium (GM) containing: Dulbecco’s modified Eagle’s high glucose (DMEM HG, Lonza) supplemented with 10% FBS (Biowest), 2.5 ng/ml basic fibroblast growth factor (bFGF, STEMCELL Technologies), penicillin (100 U/mL; Invitrogen), and streptomycin (100 µg/mL, Invitrogen) under standard conditions (37 °C, 5% CO_2_, 90% humidity). Differentiation conditions were assessed based on the literature^55^.

For adipogenic differentiation, cells were cultured for 3 days in GM, then for 3 days in an adipogenic induction medium consisting of DMEM (Lonza) with 10% FBS (Biowest), 0.5 mM 3-isobutyl-1-methylxanthine (IBMX, Sigma-Aldrich), 0.25 μM dexamethasone (Sigma-Aldrich), and 10 μg/ml insulin (PeproTech), and then for 5 days in adipogenic maintenance medium (DMEM (Lonza) with 10% FBS (Biowest) and 10 μg/ml insulin (PeproTech)). After differentiation, adipocytes were stained with an Oil Red O solution.

For fibrogenic differentiation, cells were cultured in GM for 3 days and then in GM supplemented with 1 ng/ml of TGF-β1 (PeproTech) for another 3 days followed by the alpha-smooth muscle actin (α-SMA) staining.

### Oil Red O staining for FAP-derived adipocytes

FAP-derived adipocytes were stained for lipid accumulation using lipophilic Oil Red O dye (Sigma-Aldrich). Briefly, cells were washed with PBS (Lonza), fixed in 4% PFA solution (Santa Cruz Biotechnology), washed twice with dH_2_O, and rinsed with 60% isopropanol solution (Avantor Performance Materials Poland S.A.). Meanwhile, 0.5% Oil Red O stock solution in 100% isopropanol was prepared and diluted in dH_2_O (3:2 ratio) to obtain a working solution. Subsequently, the Oil Red O solution was added to the cells for 15 min of incubation. Then, 5 washes with dH_2_O were performed. Next, pictures of the cells were taken using Nikon Eclipse Ti fluorescent microscope. Finally, the stain was extracted from cells using 100% isopropanol, and the absorbance was measured at 490 nm. Results were presented as raw absorbance values.

### α-SMA staining for FAP-derived myofibroblasts

PBS-washed cells were fixed in 4% PFA solution (Santa Cruz Biotechnology) at RT for 30 min. The cells were then permeabilized with 0.05% Triton X-100 (BioShop) in PBS (Lonza) for 5 min, washed 3 times with PBS, incubated with 0.25% glycine for 30 min, washed 3 times with PBS (Lonza) and kept in blocking solution (10% goat serum, Sigma-Aldrich) for 1 h. After overnight incubation with rabbit anti-human α-SMA (Abcam) antibody, the washing and addition of goat anti-rat Alexa Fluor 488 antibody (for detection of α-SMA) and DAPI (for detection of nuclei) was performed. The images were acquired using a Nikon Eclipse Ti or Leica DMi8 microscope with Leica DFC7000 GT fluorescent camera.

### Gene expression analysis

#### RNA isolation

TA muscles isolated from mice were immediately immersed in RNAlater® solution (Sigma-Aldrich) for stabilization of the RNA, then frozen in the nitrogen-cooled isopentane bath, and stored at − 80 °C. RNA isolation was performed according to the modified Chomczynski method^56^ with phenol–chloroform extraction. All steps were performed on ice as previously described^9,51,57^.

### Reverse transcription (RT-PCR) reaction

To synthesize cDNA, the RT-PCR reaction was carried out using 1000 ng of RNA as previously described^9,57^. The RT-PCR of miRNAs was performed with the miRCURY LNA RT Kit according to the manufacturer’s protocol (Qiagen). The samples were stored at 4 °C.

### Quantitative real-time PCR (qRT-PCR)

qRT-PCR for gene transcripts was performed with Applied Biosystems™ StepOnePlus Real-Time PCR (Thermo Fisher Scientific). The reaction mixture contained 30 ng of cDNA, Sybr® Green JumpStart™ Taq ReadyMix™ (Sigma-Aldrich), forward and reverse primers recognizing murine genes (Sigma-Aldrich) (Table 2), and nuclease-free water.

**Table 2:** The sequences of primers used for the determination of gene expression by qRT-PCR.

Gene	Sequence 5’-3’
*Col1a1*	F: CGATCCAGTACTCTCCGCTCTTCC
	R:ACTACCGGGCCGATGATGCTAACG
*Col3a1*	F: ATCTATGAATGGTGGTTTTCA
	R: TTTTGCAGTGGTATGTAATGT
*Eef2*	F: AGAACATATTATTGCTGGCG
	R: AACAGGGTCAGATTTCTTG
*Myh3*	F: TCTAGCCGGATGGTGGTCC
	R: GAATTGTCAGGAGCCACGAA
*MyoD*	F: GCTGCCTTCTACGCACCTG
	R: GCCGCTGTAATCCATCATGC
*MyoR*	F: CTTTCCAAACTGGACACGCT
	R: GCGTCCAGAGACCACGAATG
*Tgfb1*	F: GGATACCAACTATTGCTTGAG
	R: TGTCCAGGCTCCAAATATAG

For qRT-PCR for miRNAs, the reaction was carried out according to the protocol of the miRCURY LNA SYBR® Green PCR Kit (Qiagen). Briefly, the reaction mix contained: 80 × diluted cDNA template, miRCURY SYBR Green Master Mix, PCR primer mix (miRCURY LNA miRNA PCR Assays, Qiagen), and nuclease-free water. The last step included the analysis of the melt curve (60–95 °C; 0.3 °C/min). The constitutive Snord68 RNA in the case of miRNA and elongation factor 2 *(Eef2*) in the case of mRNA were used as reference genes. Relative quantification of gene expression was calculated based on the comparative cycle threshold (C_t_) method (the threshold cycle value, defined as the cycle number by which the fluorescence signal crosses the fluorescence threshold), according to the 2^–ΔCt^ formula where ΔC_t_ = C_t gene of interest_–C_t Eef2/SNORD68_) and presented as relative expression.

### Transcriptome sequencing

On day 3 after the glycerol-induced injury, FAPs were sorted according to the protocol described above. RNA from FAPs (sorted directly into lysis buffer) was isolated with the Single Cell RNA Purification Kit (Norgen Biotek Corp.) according to the manufacturer’s protocol. The initial concentration measurement was performed using a NanoDrop ND-1000 spectrophotometer (Thermo Fisher Scientific). For the experiment, 4 biological replicates from WT and 4 from miR-378^–/–^ mice were used.

The transcriptome next-generation sequencing and initial analysis were performed at the Genomics Core Facility at the Małopolska Centre of Biotechnology (MCB) using a highly multiplexed amplification method provided by the Ion AmpliSeq™ technology and the Ion Proton™ machine (Thermo Fisher Scientific). Libraries for 8 samples were prepared applying the Ion AmpliSeq™ Transcriptome Mouse Gene Expression Panel, allowing the simultaneous analysis of > 20,000 mouse RefSeq genes. Before the library preparation step, the integrity and concentration of RNA samples were determined using an Agilent 2100 Bioanalyzer with the RNA 6000 Pico Kit (Agilent Technologies). The cDNA libraries were prepared manually according to the manufacturer's protocol utilizing 10 ng of total RNA as input material. Emulsion PCR was conducted with the use of the Ion PI™ Hi-Q™ OT2 200 Kit. Eight barcoded libraries were equally pooled and sequenced on a single Ion PI™ Chip v3 chip using the Ion PI™ Hi-Q™ Sequencing 200 Kit chemistry. Primary bioinformatic analyses including quality control, mapping to the AmpliSeq_Mouse_Transcriptome_v1 reference, transcripts counting, and read-count normalization were carried out with tools and plugins available through Torrent Suite Server v5.12.1 with default parameters. Differential gene expression analysis (DGE) was carried out utilizing the DESeq2 package (with default parameters) implemented in the R version 3.3.3 software. The p-values for differentially expressed genes were corrected for multiple comparisons using the Benjamini–Hochberg approach and the results with the corrected p-values < 0.05 were considered significant. Further results were analyzed using the Search Tool for the Retrieval of Interacting Genes/Proteins (STRING) database^58^ and the Database for Annotation, Visualization and Integrated Discovery (DAVID)^59,60^.

### Protein isolation

Total protein was isolated from the frozen in nitrogen-cooled isopentane TA muscles by homogenization in the 1% Triton X-100 (BioShop) in PBS (Lonza) using TissueLyser (Qiagen). Subsequently, samples were incubated for 30 min on ice, centrifuged (14,000×*g*, 10 min, RT) and supernatants were collected and stored at − 80 °C.

### Western blot

For Fatty acid-binding protein-4 (FABP4) and phospho-SMAD family member 2 (p-SMAD2) analysis, 25 μg of protein lysates were processed as described previously^9,51^. Primary antibodies – rabbit anti-FABP4 (Cell Signalling; 2120S) and rabbit anti-pSMAD2 (Abcam; ab188334) diluted 1:500 blocking solution. Secondary antibody conjugated with horseradish peroxidase (HRP), goat anti-rabbit (1:5000; Cell Signalling). Ponceau S staining (Sigma Aldrich) was performed as a loading control. The proteins were then detected with Immobilon Western Chemiluminescent HRP Substrate (Millipore) and chemiluminescence was analyzed using the ChemiDoc Imaging System (Bio-Rad).

### Enzyme-linked immunosorbent assay (ELISA)

50 µg of protein lysates were used to detect TGF-β1 according to the vendor’s protocol (R&D Systems).

### Luminex

For the assessment of the level of IL-1β, IL-4, IL-6, IL-10, IL-13, IGF-1, and TNF-α, 1:5 dilution (in Calibrator Diluent) of the tissue protein lysates (TA muscle w/o glycerol injection and in all tested time points: 1, 3, 7, 14, and 28 days after injury) and 1:3 dilution (in Calibrator Diluent) of the cell medium collected from above WT and miR-378^–/–^ FAPs cells after 4 days of culture were subjected to the Mouse Luminex® Discovery Assay (R&D Systems) according to the manufacturer’s protocol. Measurement was carried out using Luminex® 200™. The results were calculated as pg/mg of total protein for tissue lysates and mg/ml for cell medium.

### Statistical analyses

Data are presented as mean ± SEM and evaluated with the unpaired two-tailed Student’s *t* test to determine differences between two groups (when mentioned) or 1-way ANOVA followed by Tukey’s post hoc test for multiple groups. The results were counted as significant at a value of p < 0.05. Grubb’s test was used to identify significant outliers while GraphPad Prism 8 for graphs and statistical analyses.

### Supplementary Information

Supplementary Information.

## Data Availability

The RNA sequencing data were deposited in the NCBI Sequence Read Archive (SRA) (BioProject accession numbers: PRJNA944138). Source data for the graphs and charts are provided as Supplementary Data and in Supplementary Table 1. Uncropped blot images were included as Supplementary Fig. 6. All other data are available from the corresponding author on reasonable request.
