# Supplementary material for: miR-378 influences muscle satellite cells and enhances adipogenic potential of fibro-adipogenic progenitors but does not affect muscle regeneration in the glycerol-induced injury model
Source: Sci Rep. 2023 Aug 18;13:13434. doi: 10.1038/s41598-023-40729-x (PMC10439181; doi:10.1038/s41598-023-40729-x)
Supplement: Supplementary file 1 — Supplementary Information. [file 41598_2023_40729_MOESM1_ESM.pdf]

# miR-378 influences muscle satellite cells and enhances adipogenic potential of fibro-adipogenic progenitors but does not affect muscle regeneration in the glycerol-induced injury model

Olga Mucha[1], Paulina Podkalicka[1], Monika Żukowska[1], Ewelina Pośpiech[2], Józef Dulak[1], Agnieszka Łoboda[1]*

[1]*Department of Medical Biotechnology, Faculty of Biochemistry, Biophysics and Biotechnology, Jagiellonian University in Krakow, Kraków, 30-387, Poland.*
[2]*Malopolska Centre of Biotechnology in Krakow, Kraków, 30-387, Poland*

* *Corresponding author*
Agnieszka Łoboda, PhD, DSc; Department of Medical Biotechnology; Faculty of Biochemistry, Biophysics and Biotechnology; Jagiellonian University; Gronostajowa 7, 30-387, Krakow, Poland; email: agnieszka.loboda@uj.edu.pl; phone: +48 12 664 6412.

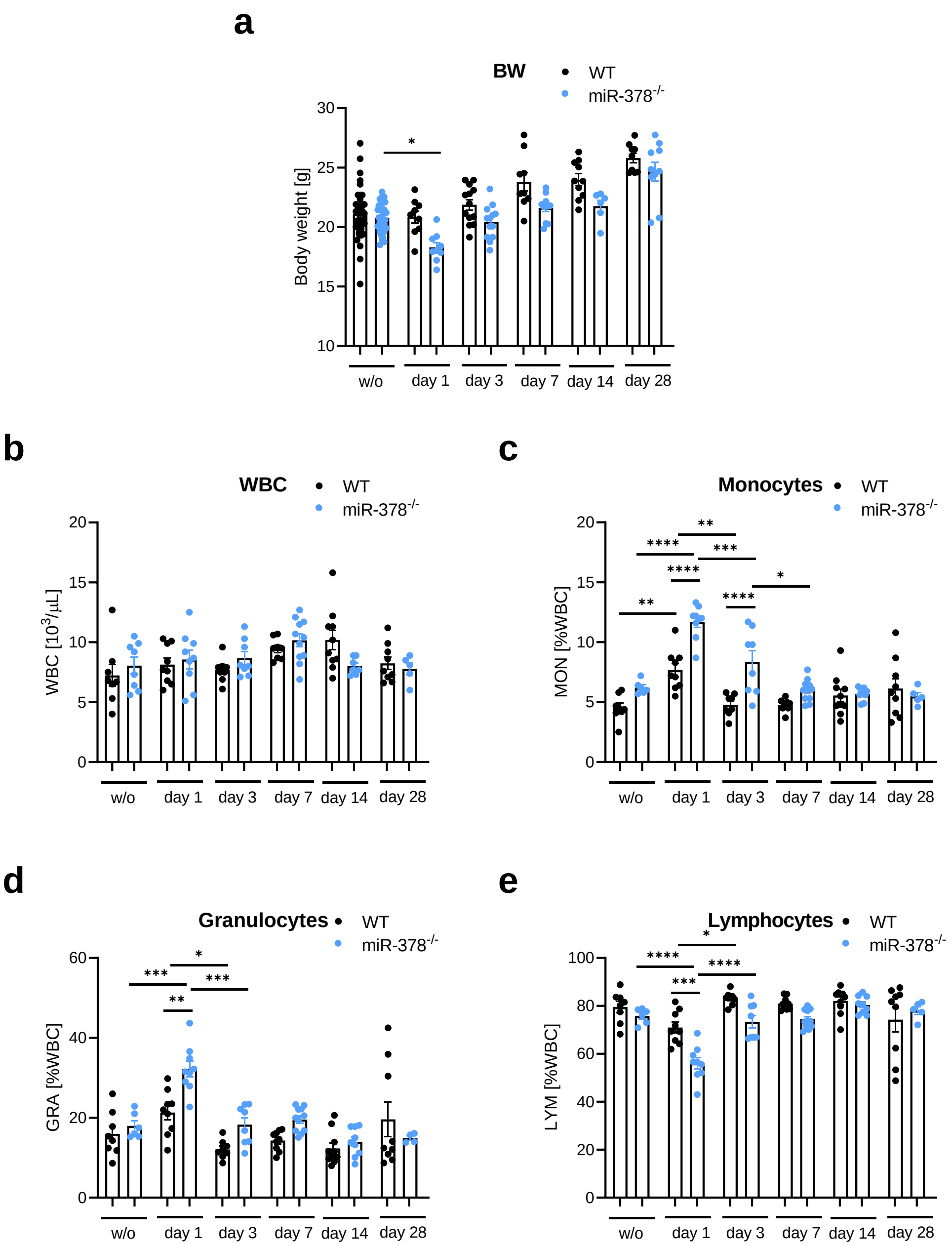


**Supplementary Figure 1.** miR-378 deletion affects the percentage of monocytes, granulocytes, and lymphocytes during the early stage of muscle regeneration. (**a**) miR-378 deletion has no severe impact on the body weight (BW) of the mice during muscle regeneration. Data are presented as mean ± SEM; n=6–36/group; (**b**) The unchanged number of white blood cells (WBC) in the peripheral blood; n=5–11/group. Increased percentage of (**c**) monocytes and (**d**) granulocytes in miR-378$^{-/-}$ mice on day 1 after the injection. (**e**) Decreased percentage of lymphocytes in miR-378$^{-/-}$ animals on day 1 in comparison to WT counterparts. Results shown as a mean ± SEM; *p < 0.05; **p < 0.01; ***p < 0.001; ****p < 0.0001 by 1-way ANOVA test with Tukey's post hoc test.

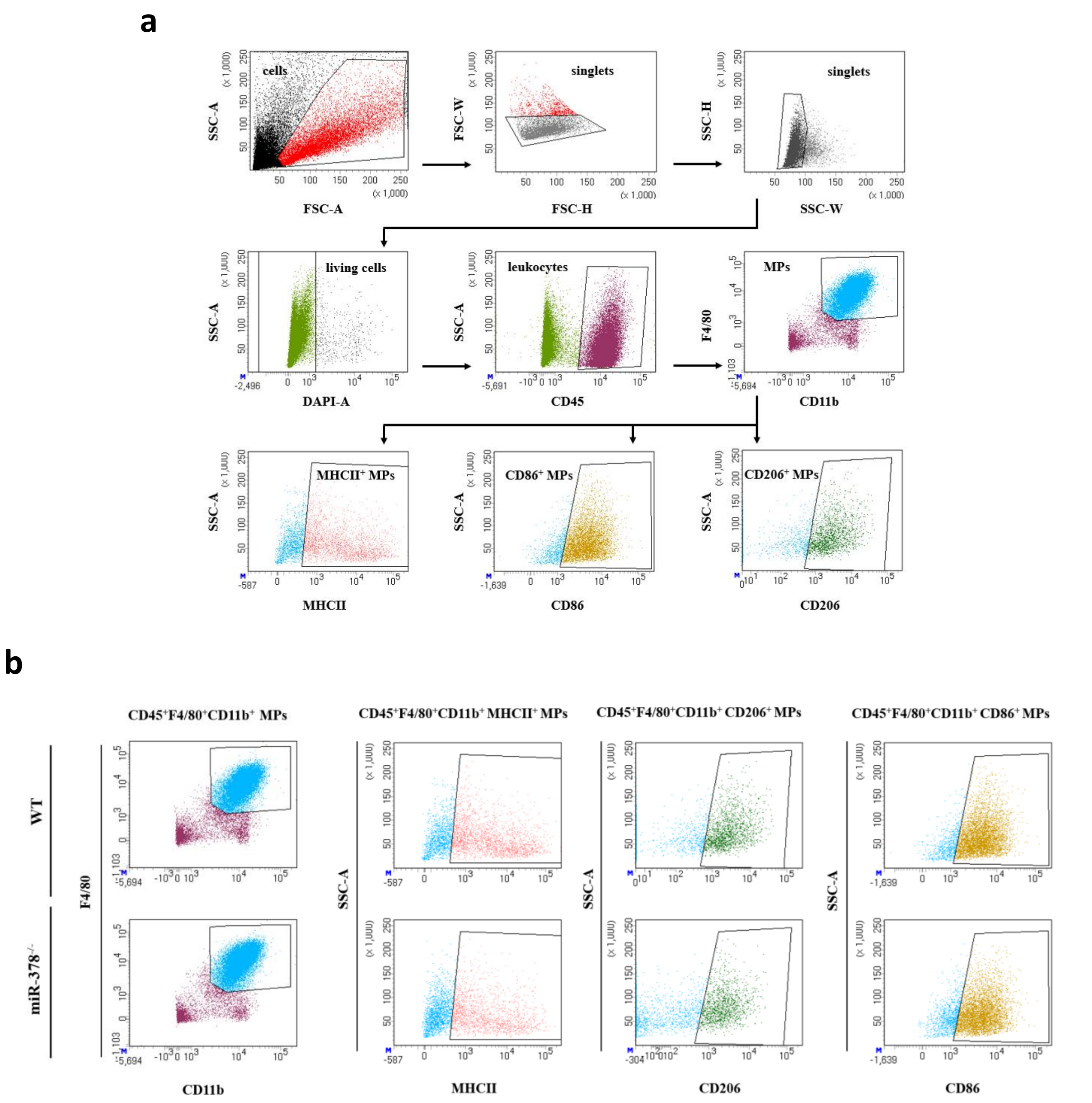


**Supplementary Figure 2.** Gating strategy for (**a**) macrophages and (**b**) shortened gaiting strategy for various populations of macrophages.

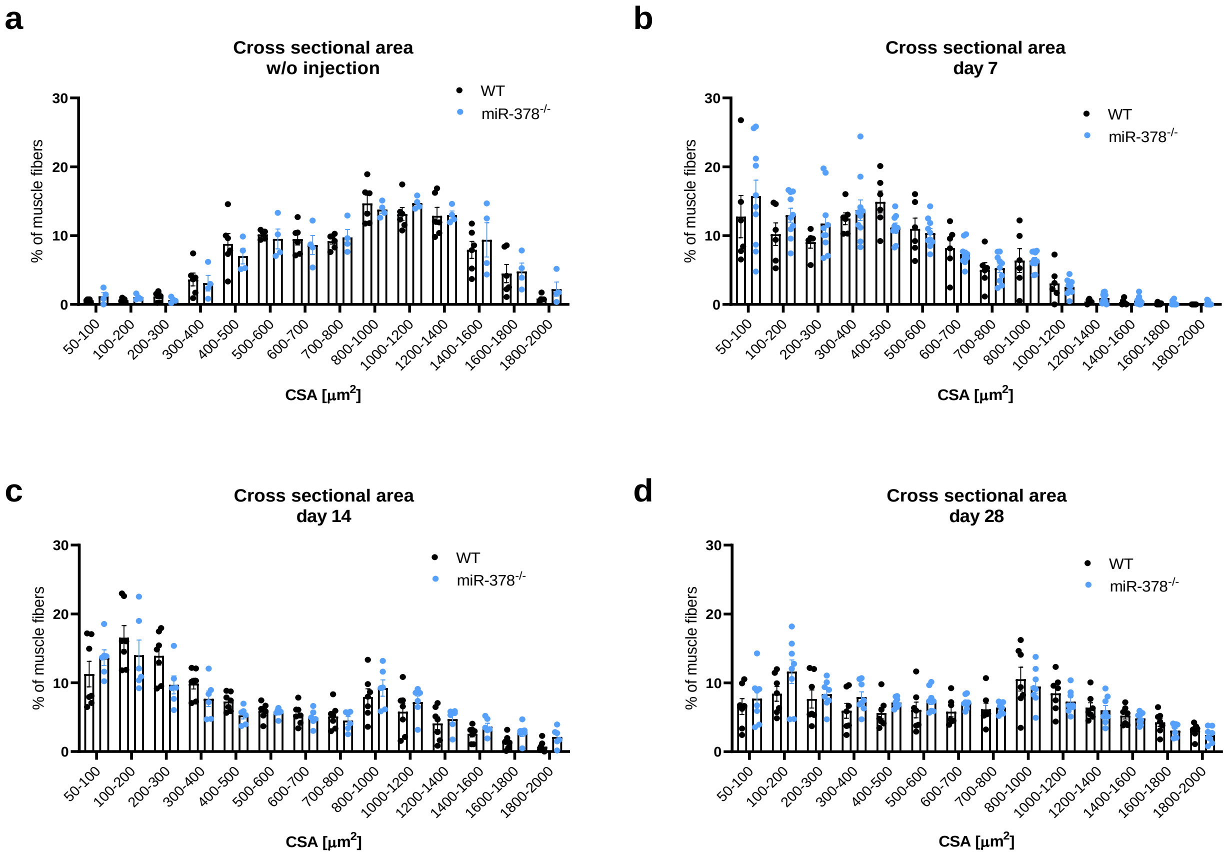


**Supplementary Figure 3.** No changes between genotypes in cross-sectional area (CSA) (**a**) without injury (n= 4-6/group), on day (**b**) 7 (n= 6-10/group), (**c**) 14 (n= 6-7/group), and (**d**) 28 (n= 7-8/group) after injury were observed. Results shown as mean ± SEM.

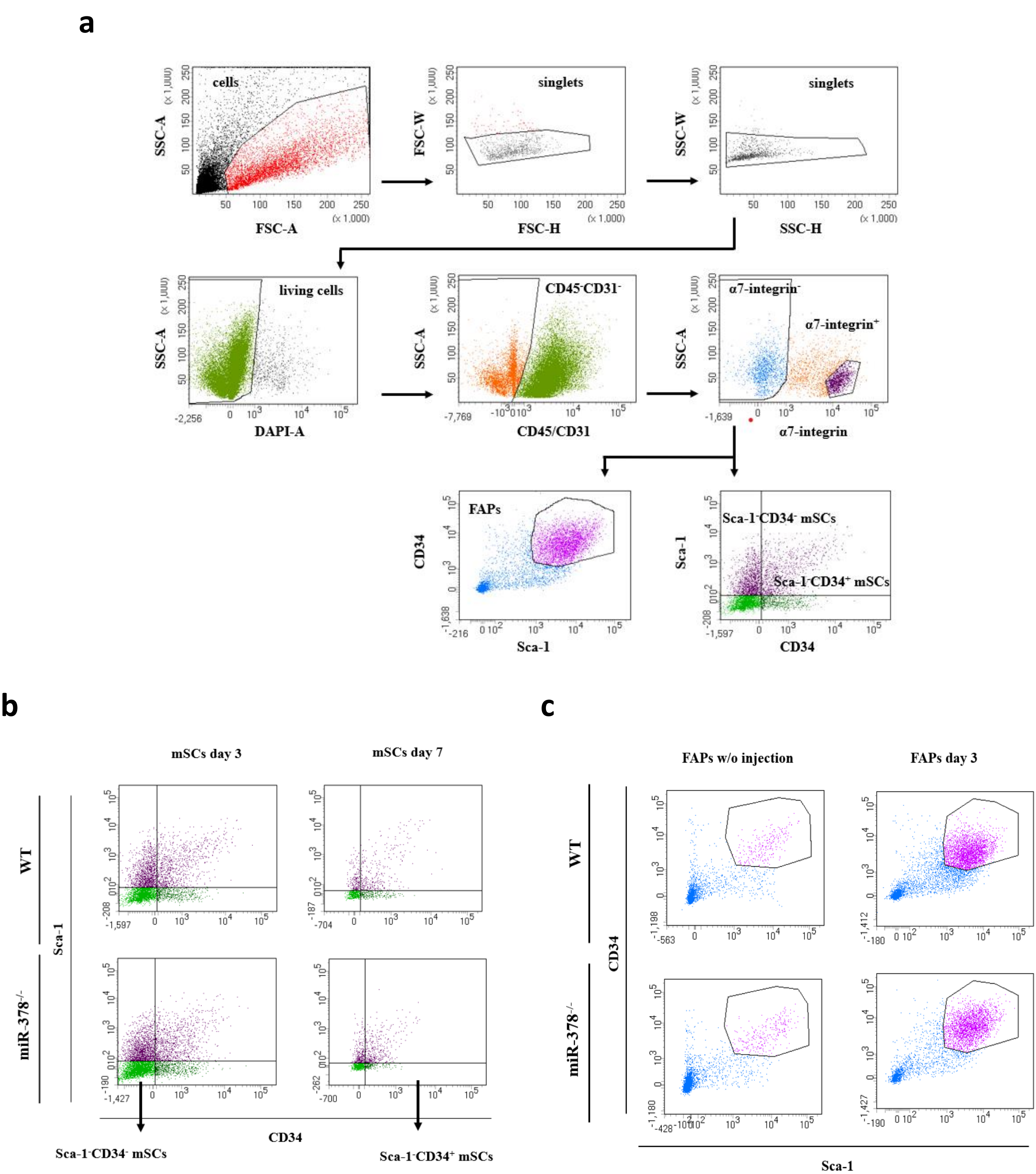


**Supplementary Figure 4.** Gating strategy for (**a**) fibro-adipogenic progenitors (FAPs) together with muscle satellite cells (mSCs). Shortened gating strategy for (**b**) mSCs and (**c**) FAPs on various days after injury or uninjured muscle.

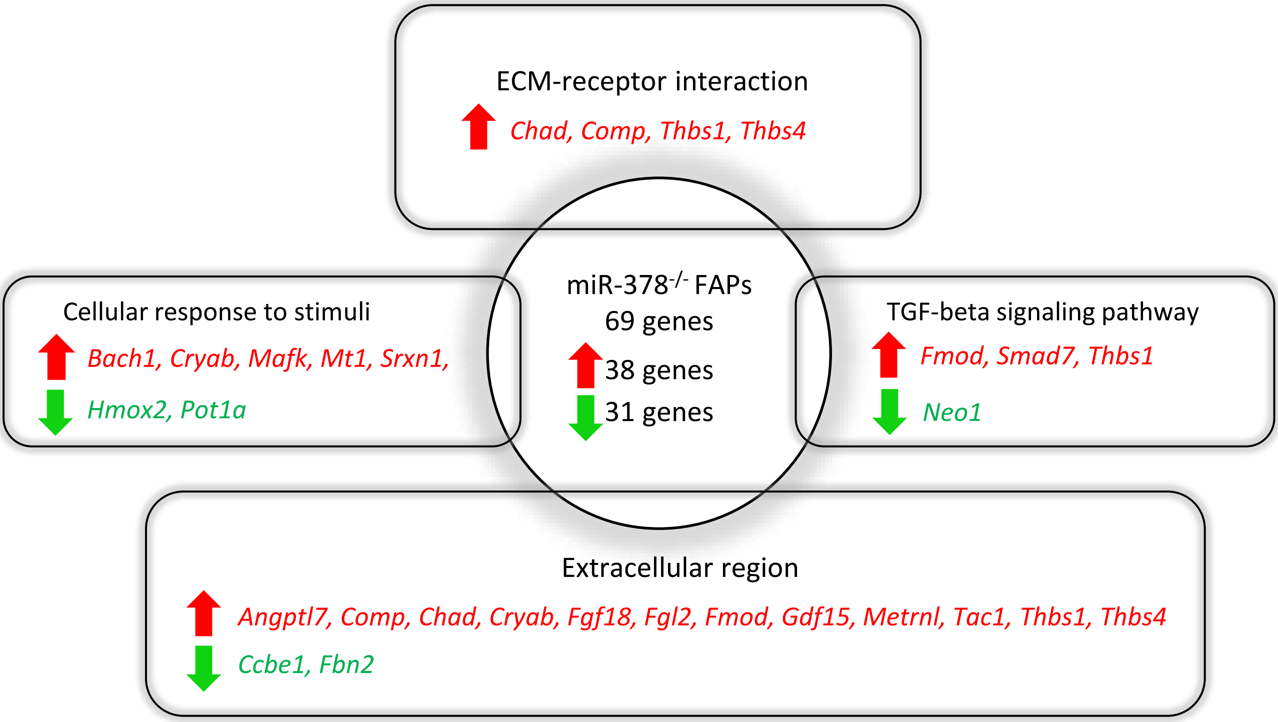


**Supplementary Figure 5.** Basic analysis using the Database for Annotation, Visualization and Integrated Discovery (DAVID) database showing examples of processes which were significantly enriched based on transcriptomic analysis of WT and miR-378$^{-/-}$ FAP cells. Red arrows show how many genes were upregulated in miR-378$^{-/-}$ mice. Green arrows show how many genes were downregulated in miR-378$^{-/-}$ mice, $p_{adj}$ value < 0.1 (p value <0.0008).

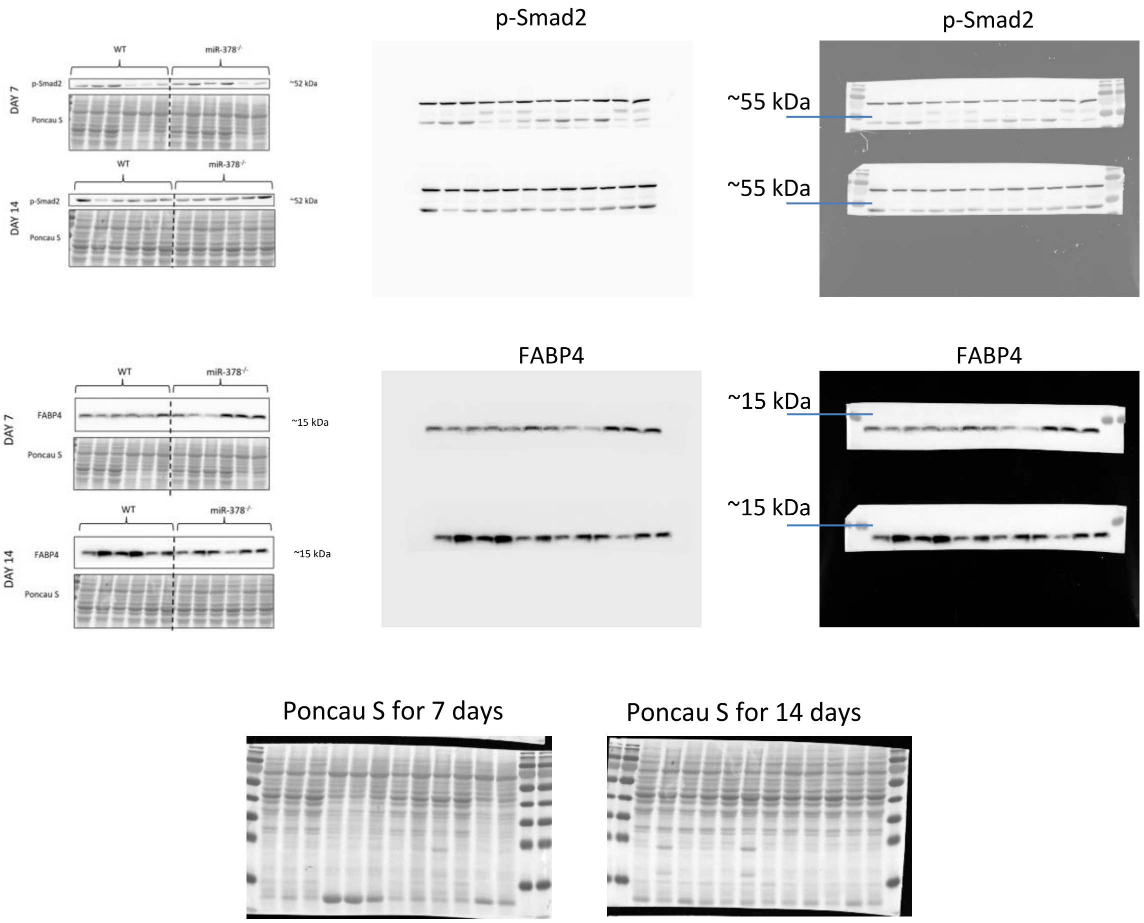


**Supplementary Figure 6.** Uncropped blot images

**Supplementary Table 1**. List of the changed genes from transcriptomic analysis (miR-378$^{-/-}$ FAPs vs. WT FAPs) sorted based on the $p_{adj}$ value. Threshold: $p_{adj} < 0.1$.

| Gene name | Base Mean | log2 Fold Change | pvalue | padj | Fold Change |
|---|---|---|---|---|---|
| ***Fbn2*** | 711.41 | -5.10 | 6.85E-175 | 5.81E-171 | 0.03 |
| ***Aldh7a1*** | 736.45 | -2.06 | 1.03E-56 | 4.36E-53 | 0.24 |
| ***Nol12*** | 190.87 | 0.88 | 3.73E-07 | 1.05E-03 | 1.84 |
| ***Cryab*** | 681.90 | 0.74 | 4.99E-07 | 1.06E-03 | 1.67 |
| ***Fmod*** | 2304.62 | 0.61 | 9.19E-07 | 1.56E-03 | 1.53 |
| ***Hist1h1c*** | 1379.77 | 0.60 | 5.54E-06 | 7.84E-03 | 1.52 |
| ***Hdac2*** | 1815.92 | -0.57 | 6.49E-06 | 7.87E-03 | 0.68 |
| ***Ugdh*** | 4392.13 | 0.64 | 1.71E-05 | 1.63E-02 | 1.56 |
| ***Mndal*** | 716.84 | -0.63 | 1.73E-05 | 1.63E-02 | 0.65 |
| ***Metrnl*** | 1208.76 | 0.72 | 3.38E-05 | 2.58E-02 | 1.65 |
| ***Gdf15*** | 238.49 | 0.75 | 3.38E-05 | 2.58E-02 | 1.68 |
| ***Hmox2*** | 1167.77 | -0.68 | 3.65E-05 | 2.58E-02 | 0.63 |
| ***Slc3a2*** | 1975.19 | 0.60 | 4.00E-05 | 2.61E-02 | 1.52 |
| ***Mt1*** | 13736.25 | 0.66 | 4.81E-05 | 2.91E-02 | 1.58 |
| ***Acaa2*** | 971.94 | 0.55 | 6.93E-05 | 3.72E-02 | 1.46 |
| ***Chad*** | 176.44 | 0.74 | 7.01E-05 | 3.72E-02 | 1.67 |
| ***Bach1*** | 1716.44 | 0.59 | 7.66E-05 | 3.72E-02 | 1.51 |
| ***Aff1*** | 1801.84 | 0.47 | 7.90E-05 | 3.72E-02 | 1.39 |
| ***Gm6548*** | 324.39 | -0.62 | 9.24E-05 | 3.86E-02 | 0.65 |
| ***Smim3*** | 1521.28 | 0.48 | 9.37E-05 | 3.86E-02 | 1.40 |
| ***Ccbe1*** | 1707.34 | -0.46 | 9.54E-05 | 3.86E-02 | 0.73 |
| ***Angel2*** | 713.11 | -0.61 | 1.03E-04 | 3.97E-02 | 0.65 |
| ***Parg*** | 490.43 | -0.58 | 1.10E-04 | 4.05E-02 | 0.67 |
| ***Rfx2*** | 178.71 | 0.68 | 1.22E-04 | 4.20E-02 | 1.61 |
| ***Herc4*** | 449.68 | -0.57 | 1.24E-04 | 4.20E-02 | 0.67 |
| ***Angptl7*** | 488.69 | 0.74 | 1.42E-04 | 4.50E-02 | 1.67 |
| ***Thbs4*** | 9390.08 | 0.68 | 1.44E-04 | 4.50E-02 | 1.60 |
| ***Slc35a3*** | 178.09 | -0.56 | 1.48E-04 | 4.50E-02 | 0.68 |
| ***Srxn1*** | 1506.59 | 0.60 | 1.64E-04 | 4.76E-02 | 1.51 |
| ***Dcaf10*** | 576.25 | -0.53 | 1.68E-04 | 4.76E-02 | 0.69 |
| ***Klf4*** | 4197.05 | 0.64 | 1.76E-04 | 4.81E-02 | 1.56 |
| ***Setx*** | 891.07 | -0.62 | 2.06E-04 | 5.31E-02 | 0.65 |
| ***Fgl2*** | 926.11 | 0.69 | 2.06E-04 | 5.31E-02 | 1.61 |
| ***Gas1*** | 1419.50 | -0.50 | 2.25E-04 | 5.51E-02 | 0.71 |
| ***Rpgrip1l*** | 285.57 | -0.62 | 2.27E-04 | 5.51E-02 | 0.65 |
| ***Myo9a*** | 515.82 | -0.48 | 2.66E-04 | 6.03E-02 | 0.72 |
| ***Dusp5*** | 797.48 | 0.53 | 2.66E-04 | 6.03E-02 | 1.44 |

| | | | | | |
|---|---|---|---|---|---|
| ***Neto2*** | 113.63 | -0.69 | 2.70E-04 | 6.03E-02 | 0.62 |
| ***Snx6*** | 1294.64 | -0.62 | 2.87E-04 | 6.07E-02 | 0.65 |
| ***0610038B21Rik*** | 134.47 | -0.57 | 2.90E-04 | 6.07E-02 | 0.67 |
| ***Slc30a9*** | 398.34 | -0.64 | 2.93E-04 | 6.07E-02 | 0.64 |
| ***Phf14*** | 430.27 | -0.52 | 3.33E-04 | 6.72E-02 | 0.70 |
| ***Nectin1*** | 691.82 | 0.47 | 3.46E-04 | 6.83E-02 | 1.38 |
| ***Pdlim4*** | 582.53 | 0.56 | 3.58E-04 | 6.85E-02 | 1.47 |
| ***Comp*** | 710.42 | 0.51 | 3.63E-04 | 6.85E-02 | 1.42 |
| ***Uqcrq*** | 901.67 | 0.54 | 3.74E-04 | 6.91E-02 | 1.45 |
| ***Nisch*** | 3661.46 | -0.56 | 3.99E-04 | 7.21E-02 | 0.68 |
| ***Fat1*** | 1490.28 | 0.44 | 4.21E-04 | 7.45E-02 | 1.36 |
| ***Klf2*** | 3421.11 | 0.43 | 5.00E-04 | 8.59E-02 | 1.35 |
| ***Has1*** | 12670.76 | 0.49 | 5.06E-04 | 8.59E-02 | 1.41 |
| ***Mafk*** | 943.32 | 0.43 | 5.43E-04 | 8.83E-02 | 1.35 |
| ***Ndn*** | 656.25 | -0.48 | 5.53E-04 | 8.83E-02 | 0.72 |
| ***Snx19*** | 635.72 | -0.41 | 5.54E-04 | 8.83E-02 | 0.75 |
| ***Hist2h4*** | 2583.09 | 0.40 | 5.62E-04 | 8.83E-02 | 1.32 |
| ***Thbs1*** | 23153.45 | 0.40 | 5.98E-04 | 9.09E-02 | 1.32 |
| ***Spats2l*** | 322.32 | -0.61 | 6.00E-04 | 9.09E-02 | 0.66 |
| ***Hk2*** | 5204.01 | 0.39 | 6.30E-04 | 9.14E-02 | 1.31 |
| ***Fgf18*** | 224.53 | 0.62 | 6.34E-04 | 9.14E-02 | 1.54 |
| ***Ckap4*** | 4031.40 | 0.58 | 6.36E-04 | 9.14E-02 | 1.49 |
| ***Pot1a*** | 174.44 | -0.54 | 6.85E-04 | 9.29E-02 | 0.69 |
| ***Neo1*** | 2031.12 | -0.47 | 6.87E-04 | 9.29E-02 | 0.72 |
| ***Ttk*** | 102.44 | -0.63 | 6.95E-04 | 9.29E-02 | 0.65 |
| ***Smad7*** | 2138.98 | 0.46 | 6.98E-04 | 9.29E-02 | 1.37 |
| ***Ndrg1*** | 2080.14 | 0.42 | 7.01E-04 | 9.29E-02 | 1.34 |
| ***Nnmt*** | 937.65 | 0.51 | 7.39E-04 | 9.64E-02 | 1.43 |
| ***Wdr19*** | 163.89 | -0.54 | 7.86E-04 | 9.98E-02 | 0.69 |
| ***Tac1*** | 208.31 | 0.55 | 8.04E-04 | 9.98E-02 | 1.46 |
| ***Ubr1*** | 577.49 | -0.50 | 8.09E-04 | 9.98E-02 | 0.71 |
| ***Cyld*** | 214.92 | -0.55 | 8.12E-04 | 9.98E-02 | 0.68 |
